# Noncovalent Immobilization of Pentamethylcyclopentadienyl Iridium Complexes on Ordered Mesoporous Carbon for Electrocatalytic Water Oxidation

**DOI:** 10.1002/smsc.202100037

**Published:** 2021-08-06

**Authors:** Ana M. Geer, Chang Liu, Charles B. Musgrave, Christopher Webber, Grayson Johnson, Hua Zhou, Cheng-Jun Sun, Diane A. Dickie, William A. Goddard, Sen Zhang, T. Brent Gunnoe

**Affiliations:** ^1^ Department of Chemistry University of Virginia Charlottesville VA 22904 USA; ^2^ Materials and Process Simulation Center Department of Chemistry California Institute of Technology Pasadena CA 91125 USA; ^3^ Advanced Photon Source Argonne National Laboratory Lemont IL 60439 USA

**Keywords:** electrocatalysis, iridium molecular complexes, noncovalent immobilization, ordered mesoporous carbon, water oxidation

## Abstract

The attachment of molecular catalysts to conductive supports for the preparation of solid‐state anodes is important for the development of devices for electrocatalytic water oxidation. The preparation and characterization of three molecular cyclopentadienyl iridium(III) complexes, Cp*Ir(1‐pyrenyl(2‐pyridyl)ethanolate‐κO,κN)Cl (**1**) (Cp* = pentamethylcyclopentadienyl), Cp*Ir(diphenyl(2‐pyridyl)methanolate‐κO,κN)Cl (**2**), and [Cp*Ir(4‐(1‐pyrenyl)‐2,2′‐bipyridine)Cl]Cl (**3**), as precursors for electrochemical water oxidation catalysts, are reported. These complexes contain aromatic groups that can be attached via noncovalent π‐stacking to ordered mesoporous carbon (OMC). The resulting iridium‐based OMC materials (**Ir‐1**, **Ir‐2,** and **Ir‐3**) were tested for electrocatalytic water oxidation leading to turnover frequencies (TOFs) of 0.9–1.6 s^−1^ at an overpotential of 300 mV under acidic conditions. The stability of the materials is demonstrated by electrochemical cycling and X‐ray absorption spectroscopy analysis before and after catalysis. Theoretical studies on the interactions between the molecular complexes and the OMC support provide insight onto the noncovalent binding and are in agreement with the experimental loadings.

## Graphical abstract

Ir‐based solid‐state anodes are prepared via π‐stacking of cyclopentadienyl iridium(III) complexes onto ordered mesoporous carbon. The Ir materials are found to catalyze electrochemical water oxidation under acidic conditions exhibiting high turnover frequencies at relatively low overpotentials. Moreover, X‐ray absorption spectroscopy (XAS) confirms the Ir complexes that retain atomic‐site catalytic centers after electrochemical reactivity.

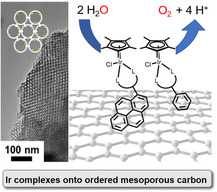

## 1 Introduction

To transform the current energy landscape, it is increasingly important to find a clean, renewable energy source to replace fossil fuels.^[^ ^1^ ^]^ Artificial photosynthesis by water splitting has been proposed as a promising alternative, and one approach is to use electrical power produced from renewable energy sources to split water into dihydrogen.^[^ ^2^, ^3^, ^4^ ^]^ The catalytic oxygen evolution half‐reaction (OER) is necessary for overall water splitting. Therefore, efficient and stable water oxidation catalysts that can convert water to dioxygen at relatively low overpotentials are a key focal point for water splitting devices. But, electrocatalytic water oxidation presents several challenges including commonly observed sluggish kinetics and catalyst instability.^[^ ^5^, ^6^, ^7^, ^8^ ^]^ Therefore, advances for solid‐state electroanodes that could be used in the assembly of artificial photosynthesis devices are desirable for the development of water‐splitting devices.

**Scheme 1:**
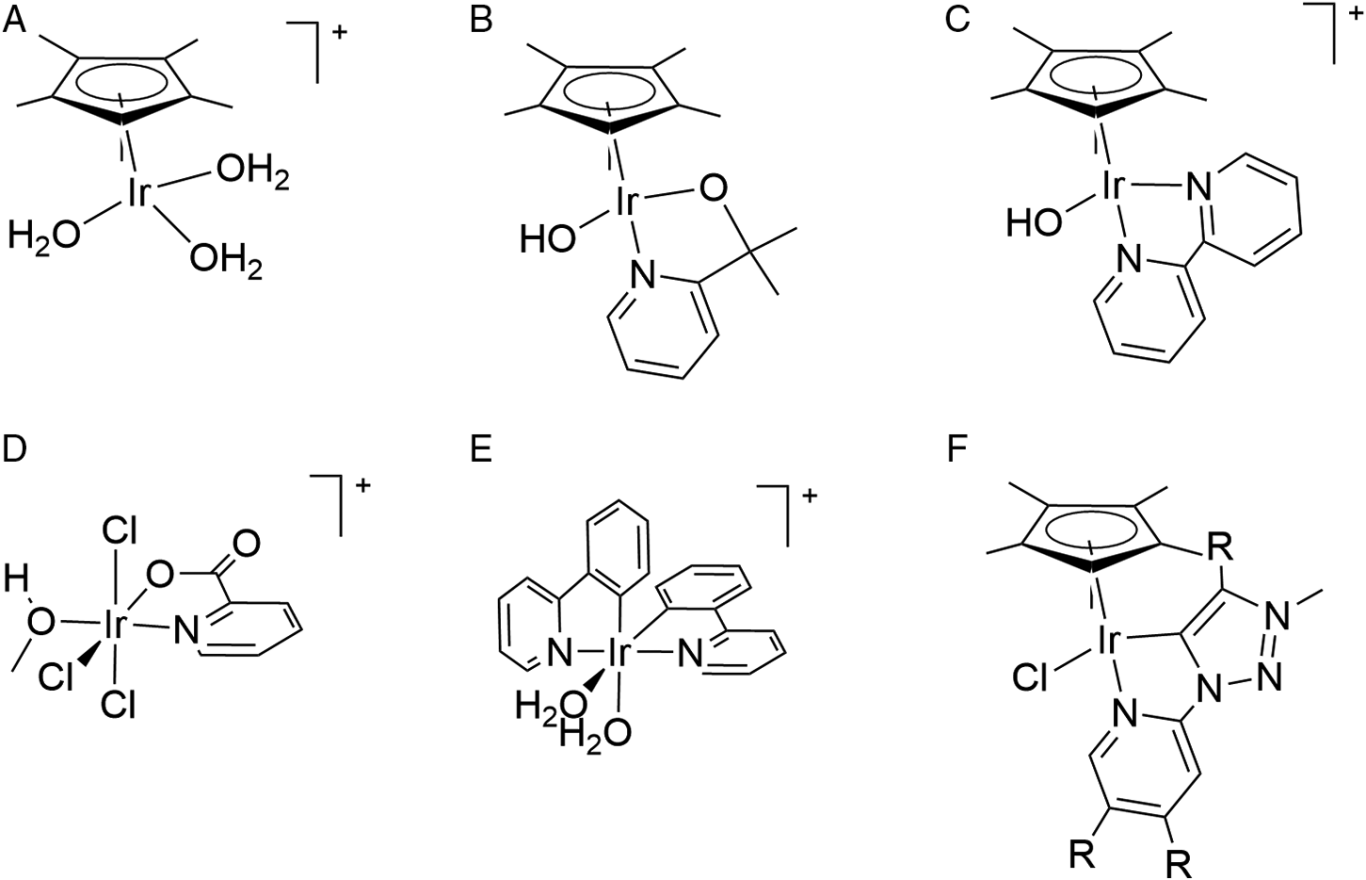
Structures of molecular iridium complexes previously studied as catalyst precursors for electrochemical water oxidation. A,B) the study by Schley et al.^[^ ^29^ ^]^; C) the study by Thomsen et al.^[^ ^28^ ^]^; D) the study by Abril et al.,^[^ ^27^ ^]^ E) the study by van Dijk et al.^[^ ^26^ ^]^; F) the study by Olivares et al.^[^ ^25^ ^]^

Among the most efficient molecular water oxidation catalysts are complexes based on Ru and Ir.^[^ ^6^, ^9^, ^10^ ^]^ There are a large number of homogeneous Ir precatalysts for catalytic water oxidation. Many leading efforts to study these molecular Ir catalysts have focused on the use of chemical oxidants (e.g., NaIO_4_ and ceric ammonium nitrate),^[^ ^9^, ^11^, ^12^, ^13^, ^14^, ^15^, ^16^, ^17^, ^18^, ^19^, ^20^, ^21^, ^22^, ^23^, ^24^ ^]^ with perhaps fewer studies on electrochemically driven water oxidation.^[^ ^25^, ^26^, ^27^, ^28^, ^29^ ^]^ For example, Crabtree and coworkers identified the tris‐aqua complex [Cp*Ir(H_2_O)_3_]_2_ (**A**) (Cp* = pentamethylcyclopentadienyl) and the complex bearing the 2‐(2‐pyridyl)‐2‐propanolate ligand, **B**, as molecular water oxidation catalyst precursors at pH 7 and 1.7 V versus normal hydrogen electrode (NHE) (**Scheme** **1**). They demonstrated that complex **A** is likely a precursor to a heterogeneous Ir catalyst.^[^ ^29^ ^]^ In a follow‐up study, the electrocatalytic activities using complexes **A**, **B,** and **C** after activation with excess NaIO_4_, or by bulk electrolysis at oxidizing potentials (≥1.4 V versus NHE), were compared. This study allowed the authors to propose that the oxidative activation of these Ir complexes leads to loss of the Cp* ligand, which is proposed to be necessary for O_2_ evolution.^[^ ^28^ ^]^ For the anionic iridium(III) complex **D** with a picolinate ligand, the picolinate ligand is readily lost under oxidative conditions (pH 1 at 1.9 V versus reversible hydrogen electrode [RHE]), ultimately leading to the formation of IrO_*x*_.^[^ ^27^ ^]^ In a more recent study, the Macchioni and coworkers investigated electrochemical water oxidation with a series of iridium complexes (including **A**, **D,** and **E** in Scheme 1) at potentials ≥ 1.8 V versus RHE (pH 7), finding similar activity for all of the complexes studied, and concluded that structure–activity relationships obtained with sacrificial oxidants do not necessarily translate to electrochemical conditions with factors such as electrodeposition and catalyst degradation playing a major role.^[^ ^26^ ^]^ Cp*Ir complexes with a chelating triazolylidene‐pyridyl ligand (e.g., complex **F** in Scheme 1) were tested for both electrochemical (≥1.7 V versus RHE) and chemical water oxidation. Electron‐donating groups on the triazolylidene ligand increase chemical water oxidation activity, in contrast to electrochemical oxidation where the best activity was found for the unsubstituted version.^[^ ^25^ ^]^

Homogeneous electrocatalysts often suffer from limitations including a) catalyst crossover between anode and cathode, which can be kinetically inhibiting, b) catalytic activity limited by diffusion to the electrode, and c) a lack of stability of the catalyst.^[^ ^30^ ^]^ Although, in some cases heterogeneous electrocatalysts can overcome these limitations that are often present for homogeneous catalytic systems, it is challenging to systemically tune and optimize the catalyst active sites of heterogeneous materials. The immobilization of molecular catalysts on supports for electrocatalysis is a strategy that can potentially address these drawbacks while facilitating selective water oxidation using well‐defined catalytic active sites.^[^ ^30^, ^31^, ^32^, ^33^ ^]^ For example, catalyst stability can be enhanced through immobilization by avoiding deactivation via associative intermolecular pathways. Also, enhancement of charge transfer at the electrode/catalyst interface can increase catalytic activity. Moreover, immobilization can increase the percent active catalyst by preventing soluble catalyst from leaving the electrode interface.

From an engineering perspective, it is necessary to prepare solid‐state anodes with immobilized molecular catalysts on a conductive support, and it is of importance that the resulting electrode materials can be stable to the oxidation reaction conditions. With the idea of developing materials for potential applications in electrolyzers, there have been efforts to attach molecular catalysts to conductive materials to prepare heterogenized molecular anodes. Despite reports of new materials for electrocatalytic water oxidation based on supported molecular complexes, substantial challenges and questions remain, including (but not limited to): a) What attachment strategies enhance activity and stability? b) How can the stability of the attachment be optimized to minimize catalyst leaching? c) Does the molecular structure remain intact? d) What impact does the support have on catalytic activity and mechanism?

**Scheme 2:**
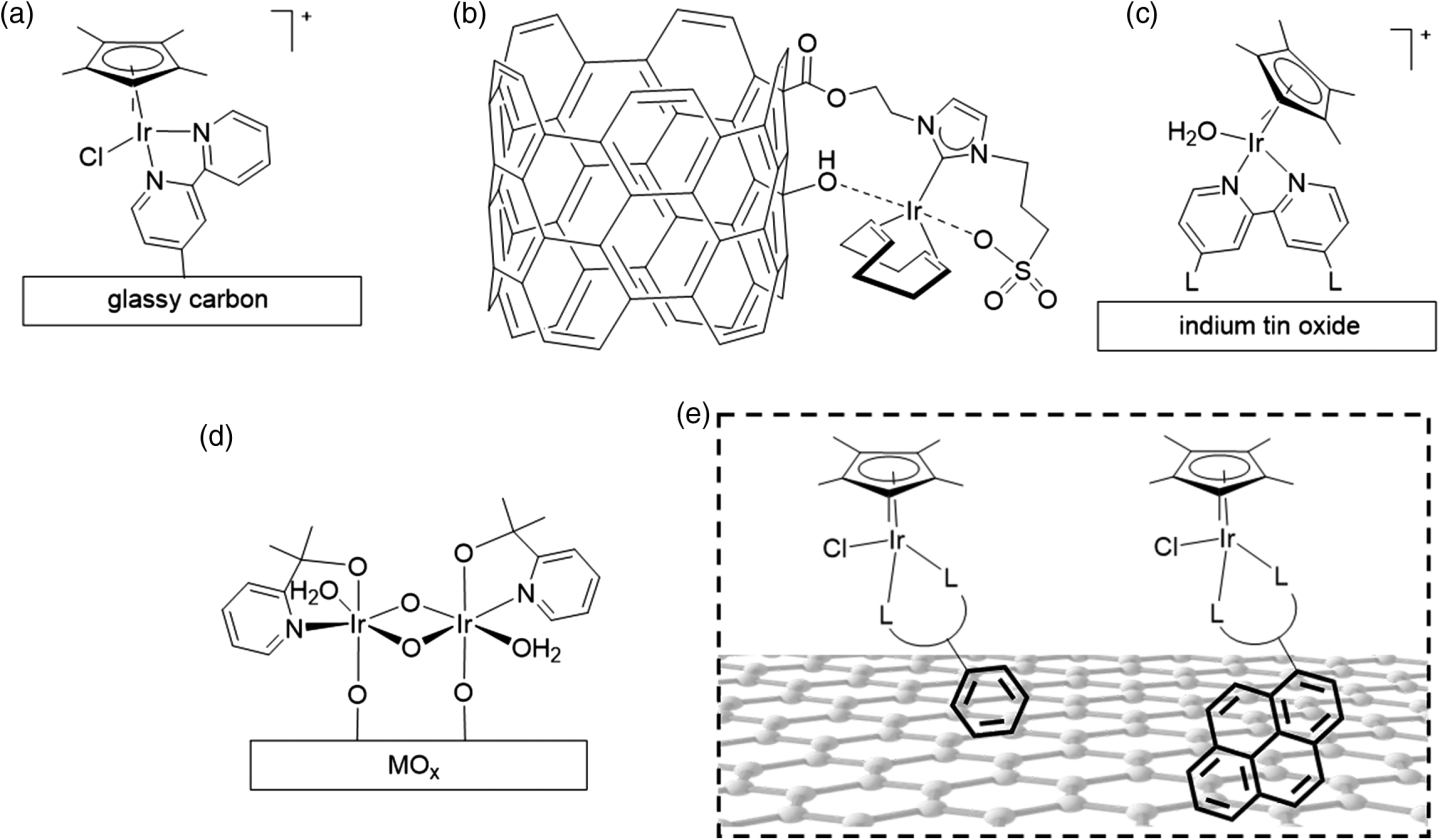
Schematic of supported iridium molecular complexes for water oxidation. a) the study by deKrafft et al. ^[^ ^35^ ^]^; b) the study by Nieto et al. and Sánchez‐Page et al.^[^ ^36^, ^37^ ^]^; c) the study by Joya et al.^[^ ^38^ ^]^; d) the study by Sheehan et al.^[^ ^41^ ^]^; e) this work.

To prepare supported molecular electrocatalysts, substantial efforts have been focused on attachment strategies that involve covalent bond formation between the molecular catalyst and the support.^[^ ^30^, ^31^, ^32^, ^34^ ^]^ Relevant examples using immobilized iridium catalysts for electrocatalytic water oxidation include covalent attachment by a diazonium grafting strategy of Cp*Ir complexes directly onto glassy carbon electrodes under slightly acidic conditions (pH = 5; **Scheme** **2**a).^[^ ^35^ ^]^ The attachment of iridium complexes to carbon nanotubes using water soluble *N*‐heterocyclic carbene ligands via ester linkage attachment for chemical oxidation in acidic conditions and electrochemical oxidation at neutral pH has been reported (Scheme 2b).^[^ ^36^, ^37^ ^]^ Cp*Ir complexes modified with carboxylate and phosphonate linkers have been covalently attached to indium tin oxide surface for electrocatalytic water oxidation at neutral and acidic pHs (Scheme 2c).^[^ ^38^ ^]^ Chemical water oxidation has been studied by immobilizing [Cp*Ir(P(O)(OH)_2_)_3_]Na on rutile TiO_2_.^[^ ^39^, ^40^ ^]^ Dinuclear iridium complexes containing pyridine alkoxide‐type ligands have been chemisorbed onto metal oxide surfaces and display high activity towards water oxidation (Scheme 2d).^[^ ^41^ ^]^ Recent achievements in noncovalent attachment strategies have also been successful for immobilization of molecular catalysts on carbon surfaces in the context of ruthenium mediated electrochemical water oxidation,^[^ ^42^, ^43^, ^44^, ^45^, ^46^ ^]^ but noncovalent supports have been less explored for iridium catalysts.^[^ ^47^ ^]^

High‐surface carbon materials, such as graphite, graphene, and carbon nanotubes, are extensively used electrocatalyst supports in oxidation reactions due to the combination of a large surface area, high corrosion and chemical resistance, and good electrical conductivity.^[^ ^48^, ^49^, ^50^ ^]^ Among them, ordered mesoporous carbon (OMC) possesses useful characteristics of large surface area, uniform pore size, and good conductivity, which makes it a promising candidate for the support material in electrochemical applications and was, therefore, chosen as a support for our iridium precatalysts.^[^ ^51^ ^]^

In this article, we have prepared Cp*Ir molecular complexes attached to OMC using π‐stacking and investigated the composite materials for electrocatalytic water oxidation under acidic conditions (Scheme 2e). In previous studies, it was found that pyrene‐modified ligands strongly attach to graphitic surfaces and can therefore increase electrocatalyst surface loadings.^[^ ^44^, ^46^, ^47^, ^49^, ^52^, ^53^, ^54^ ^]^ We selected two types of bidentate ligands with aromatic functionality, pyrenyl‐substituted bipyridine ligand and pyridine‐alkoxide type ligands with diphenyl or methyl‐pyrene substituents, with the goal of attaching the molecular complexes by π‐stacking interactions to OMC supports and comparing the π‐stacking efficacy with different chemical groups. The resulting Ir materials are efficient in electrocatalytic water oxidation in acid, exhibiting high turnover frequencies (TOFs) at relatively low overpotentials. Moreover, X‐ray absorption spectroscopy (XAS) confirmed the Ir complexes retain atomic‐site catalytic centers after electrochemical stability test. The supported electrocatalysts was also found to be stable to successive linear sweep voltammetry (LSV) cycling. Also, we used density functional theory (DFT) and Universal Force Field (UFF)^[^ ^55^ ^]^ molecular dynamics (MDs) computations and different carbon models^[^ ^56^ ^]^ to elucidate heterogenization process of Ir complexes onto OMC with noncovalent π‐stacking interactions.

## 2 Results and Discussion

### 2.1 Preparation of Molecular Iridium Complexes and Study of Potential Electrocatalytic Water Oxidation

**Scheme 3:**
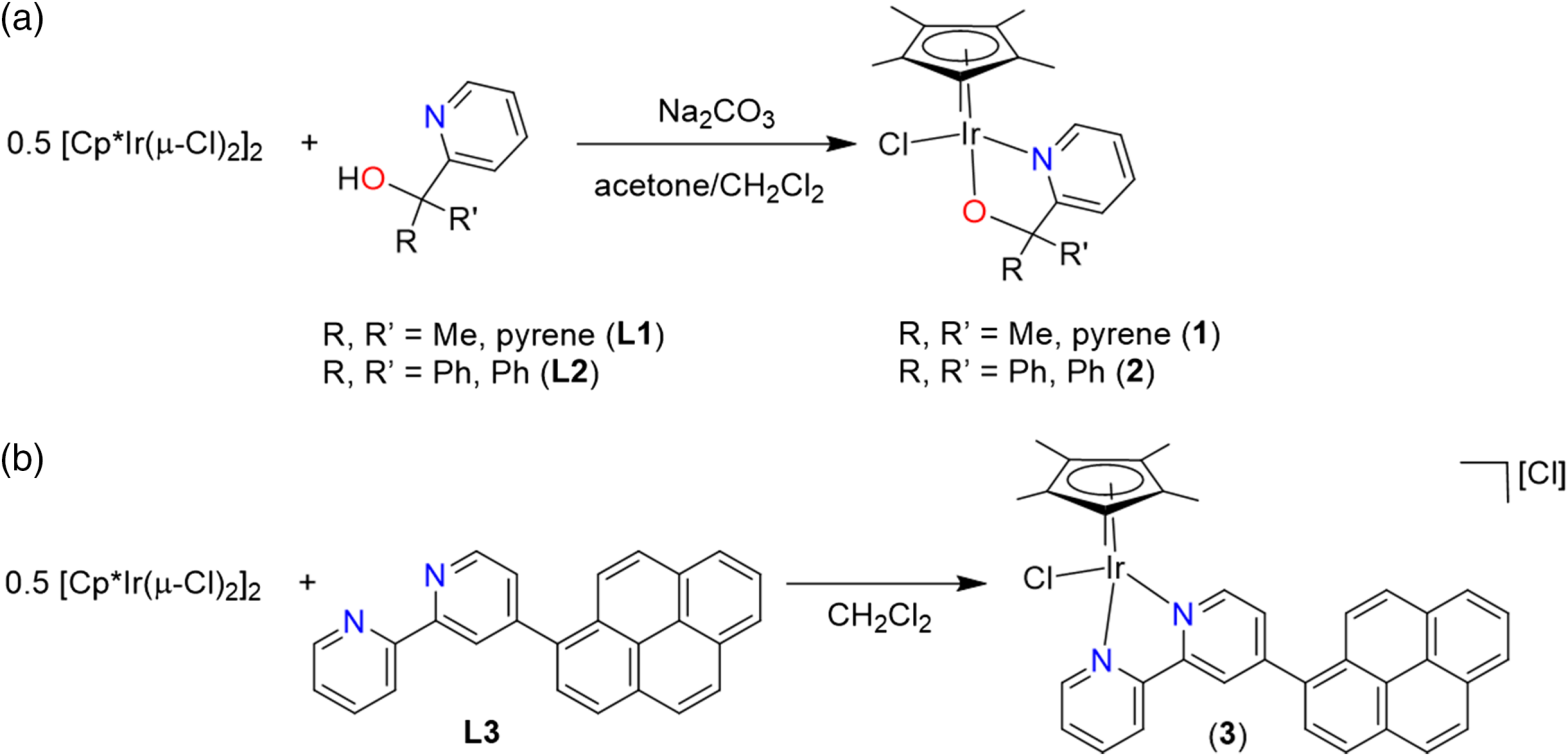
a) Synthesis of Cp*Ir(1‐pyrenyl(2‐pyridyl)ethanolate‐κO,κN)Cl (**1**) and Cp*Ir(diphenyl(2‐pyridyl)methanolate‐κO,κN)Cl (**2**). b) Synthesis of [Cp*Ir(4‐(1‐pyrenyl)‐2,2′‐bipyridine)Cl]Cl (**3**).

**Figure 1:**
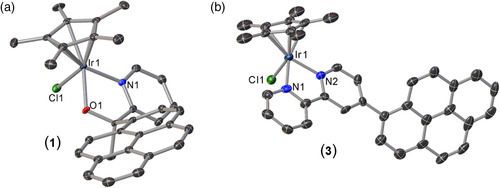
a) Oak Ridge Thermal Ellipsoid Plot (ORTEP) drawing of crystal structure of Cp*Ir(1‐pyrenyl(2‐pyridyl)ethanolate‐κO,κN)Cl (**1**) with ellipsoids shown at 50% probability. Hydrogen atoms and solvent molecules have been omitted for clarity. Selected bond lengths (Å) and angles (°) for **1**: Ir1—O1 2.0571(16), Ir1—N1 2.079(2), Ir1—Cl1 2.4508(6), Ir—Cp*(centroid) 1.7706(12), O1—Ir1—N1 77.83(7); b) ORTEP drawing of crystal structure of [Cp*Ir(4‐(1‐pyrenyl)‐2,2′‐bipyridine)Cl]Cl (**3**) with ellipsoids shown at 50% probability. Hydrogen atoms, solvent molecules, and counterions have been omitted for clarity. Selected bond lengths (Å) and angles (°) for **3**: Ir1—N1 2.077(9), Ir1—N2 2.103(8), Ir1—Cl1 2.405(3), Ir—Cp*(centroid) 1.788(8), N1—Ir1—N2 76.4(3).

The pyrene‐pyalk (pyrene‐pyalk = 1‐pyrenyl(2‐pyridyl)ethanol) ligand (**L1**) was prepared through lithiation of 2‐bromopyridine at −78 °C followed by reaction with acetylpyrene using a modified literature method.^[^ ^57^ ^]^ Cp*Ir(1‐pyrenyl(2‐pyridyl)ethanolate‐κO,κN)Cl (**1**) was prepared from the reaction of **L1** and 0.5 equivalents of [Cp*Ir(μ‐Cl)_2_]_2_ in an acetone:CH_2_Cl_2_ mixture (1:1.5) at 50 °C in the presence of excess Na_2_CO_3_ (**Scheme** **3**a). Cp*Ir(diphenyl(2‐pyridyl)methanolate‐κO,κN)Cl (**2**) was prepared as previously described (Scheme 3a),^[^ ^57^ ^]^ while the reaction between 4‐(1‐pyrenyl)‐2,2′‐bipyridine (**L3**)^[^ ^58^ ^]^ and 0.5 equivalents of [Cp*Ir(μ‐Cl)_2_]_2_ in CH_2_Cl_2_ at room temperature led to [Cp*Ir(4‐(1‐pyrenyl)‐2,2′‐bipyridine)Cl]Cl (**3**) (Scheme 3b). Complexes **1**–**3** have been characterized by NMR spectroscopy and elemental analysis (Figure S1–S6, Supporting Information). Orange crystals adequate for single‐crystal diffraction of complexes **1** and **3** were obtained by slow evaporation of CDCl_3_ solutions (**Figure** **1** and Table S1, Supporting Information). Both **1** and **3** show piano‐stool geometry around the iridium center as commonly observed for these type of complexes.^[^ ^57^, ^59^ ^]^ The solid‐state structure of **1** has comparable bond distances to the reported structure of the diphenyl derivative **2**.^[^ ^57^ ^]^ Complexes **1** and **2** have similar Ir—C distances (2.16 ± 0.02 Å), whereas the Ir—N (**1**: 2.079(2) Å; **2**: 2.089(4) Å) and Ir—O (**1**: 2.0571(16) Å; **2**: 2.064(4) Å) bond distances are slightly shorter for **1**. The solid‐state structure of **3** is very similar to the related [Cp*Ir(bpy)Cl]Cl (bpy = 2,2′‐bipyridyl),^[^ ^59^ ^]^ with similar Ir—C and Ir—Cl bond distances. The Ir1—N2 bond distance (2.103(8) Å) of **3** is slightly larger than in [Cp*Ir(bpy)Cl]Cl (Ir(1)‐N(1) = 2.076(8) Å, Ir(1)‐N(2) = 2.090(2) Å).

^1^H NMR analysis of complexes **1** and **3** in CDCl_3_ at room temperature showed the expected singlet arising from the Cp* signal at 1.17 and 1.67 ppm, respectively (Figure S3 and S5, Supporting Information). For complex **1**, the aromatic pyridine‐alkoxide ligand protons were found in the range of 10.3–6.8 ppm and displayed fluxional character in a similar manner to related [Cp*Ir(pyalk)Cl] complexes with fast ligand exchange kinetics.^[^ ^57^ ^]^ The aromatic 4‐(1‐pyrenyl)‐2,2′‐bipyridine protons in complex **3** were in the range of 9.0–7.8 ppm.^[^ ^59^ ^]^

### 2.2 Preparation of Iridium Complexes Tethered onto OMC

As a conductive carbon material, OMC has high surface area and 3D‐ordered porous texture, making it well suited to heterogeneous electrocatalysis with facilitated mass transfer. Our approach was to tether the molecular iridium complexes **1**–**3** onto OMC to form heterogenized electrocatalysts for the OER. Complexes **1**–**3** present Cp*Ir motifs that are known precatalysts for water oxidation upon oxidative removal of the Cp* ligand.^[^ ^28^, ^60^, ^61^ ^]^ Using **1**–**3**, we sought to compare the different ligand structures, pyridine‐alcohol with pyrenyl, pyridine‐alcohol without pyrenyl, and bpy with pyrenyl and efficacy for noncovalent attachment to OMC.

**Figure 2:**
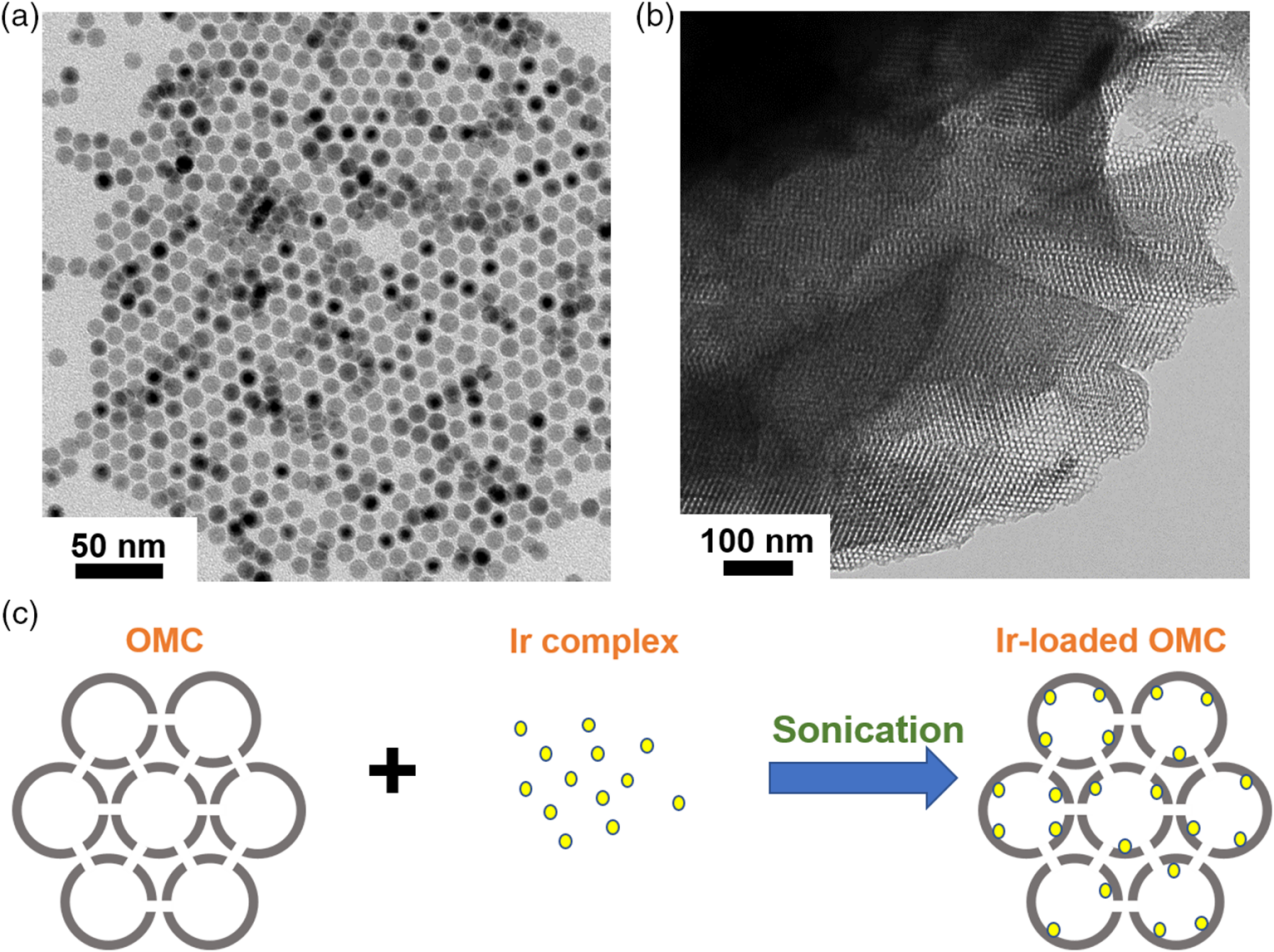
a) TEM image of Fe_3_O_4_ nanoparticles; b) TEM image of OMC annealed at 900 °C in forming gas (5% H_2_ in N_2_); c) Schematic illustration of loading process of molecular complex and OMC under sonication.

The OMC material was prepared by the carbonization of oleic acid surfactant bound on Fe_3_O_4_ nanoparticle superlattices, according to a reported method.^[^ ^51^ ^]^ In a typical synthesis, monodispersed oleic acid‐capped Fe_3_O_4_ nanoparticles with a size of 10 ± 0.5 nm were first synthesized using colloidal chemistry, as shown in transmission electron microscopy (TEM) image in **Figure** **2**a, and then assembled into a face‐centered cubic (FCC) structured nanoparticle superlattice by drying a nanoparticle solution in hexanes under ambient condition.^[^ ^51^ ^]^ The nanoparticle superlattice was subsequently treated at 500 °C under dinitrogen to carbonize the oleic acid surfactant, leading to the formation of OMC after the removal of Fe_3_O_4_ nanoparticles in an acid washing (Figure S7, Supporting Information). The resultant OMC was further annealed at 900 °C in reductive gas (5% H_2_ balanced in N_2_) to improve its graphitic degree and electrical conductivity^[^ ^51^ ^]^ and was used to immobilize the Ir complexes. As shown in Figure 2b, the OMC obtained at 900 °C exhibits a well‐defined FCC‐ordered architecture with a pore size of 7.5 nm and a wall thickness of 2.4 nm. Two prominent peaks were observed in the Raman spectrum of the isolated OMC (Figure S8, Supporting Information). The absorption at 1338 cm^−1^ corresponds to D band referring to structural defects and disorders, whereas the peak at 1594 cm^−1^ is attributed to G band arising from in‐plane vibrations of sp^2^ carbon atoms. The intensity ratio of two bands (*I*_D_/*I*_G_) is calculated to be 0.8, indicating the OMC is partially graphitic after annealing at 900 °C.^[^ ^51^ ^]^

**Figure 3:**
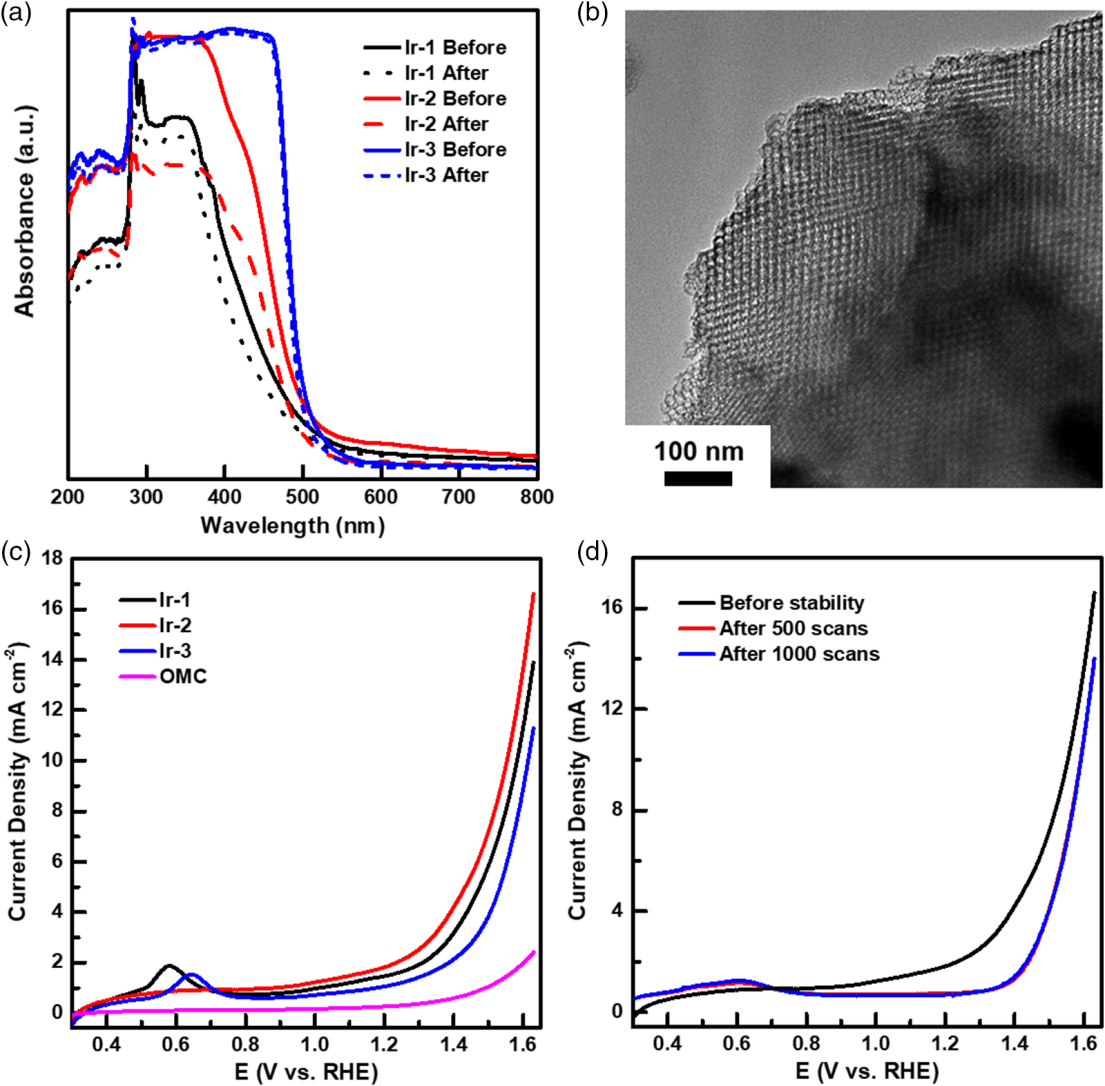
a) UV–vis absorption spectra of Ir molecular complexes before and after absorbed on OMC; b) TEM image of complex 2 loaded on OMC (**Ir‐2**); c) LSV plot of **Ir‐1**, **Ir‐2**, **Ir‐3,** and pristine OMC; d) Stability test of **Ir‐2** catalyst with continuous LSV scans.

The Ir complexes were loaded onto OMC by sonicating the mixture of OMC and complex in isopropanol for 0.5 h (Figure 2c, see Experimental Section). Our strategy is to anchor Ir complexes onto the OMC material through π‐stacking. Sonication facilitates the diffusion of molecular complexes into the interior of OMC for maximized loading density. We label the resulting Ir‐loaded OMC materials as follows: 1) Cp*Ir(1‐pyrenyl(2‐pyridyl)ethanolate‐κO,κN)Cl (**1**) on OMC is **Ir‐1**; 2) Cp*Ir{diphenyl(2‐pyridyl)methanolate‐κO,κN}Cl (**2**) on OMC is **Ir‐2**; 3) [Cp*Ir(4‐(1‐pyrenyl)‐2,2′‐bipyridine)Cl]Cl (**3**) on OMC is **Ir‐3** (Scheme 2e). The Ir‐loaded OMC materials were separated by centrifugation, and the supernatant solution containing untethered complexes was analyzed with UV–vis spectroscopy and compared with that before the addition of OMC to reveal our complex immobilization efficiency. As shown in **Figure** **3**a, the **Ir‐2** solution exhibited a more evident decrease in absorbance spectrum after loading onto OMC, compared with **Ir‐1** and **Ir‐3**, which suggests a higher loading efficiency of **Ir‐2** in our method. Further quantitative inductively coupled plasma optical emission spectrometry (ICP‐OES) analysis on complex‐tethered OMC samples showed that Ir loadings on OMC are 1.91, 2.95, and 1.17% for **Ir‐1**, **Ir‐2,** and **Ir‐3**, respectively, which is consistent with UV–vis results.

### 2.3 Theoretical Analysis of Loading Efficiency for Ir Complexes

Computational methods were utilized to understand the varying loading performances of the three Ir complexes **1**–**3** using DFT and universal force field (UFF)^[^ ^55^ ^]^ MDs. The binding of the Ir complexes to carbon supports was evaluated at two separate levels. First the binding of the Ir complexes to a periodic sheet of graphene was modeled. Afterward, the investigation of binding to the surface of low‐density (2.48 g cc^−1^) amorphous carbon (LDAC) was conducted.^[^ ^56^ ^]^ We expect that the LDAC is a good model to represent the partially graphitic OMC with nonflat surface.

**Figure 4:**
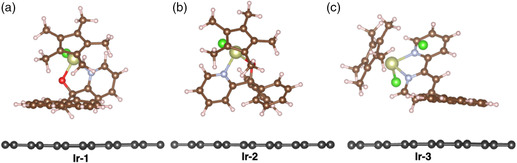
PBE—D3‐predicted binding of the Ir complexes (numbered) to a periodic sheet of 6 × 6 graphene (dark gray atoms). Periodic images extend infinitely in the *x* and *y* directions. A vacuum was placed above the Ir complexes to inhibit interaction of the periodic images in the *z* direction.

Hybrid DFT (B3LYP—D3) was used to investigate the binding of the Ir complexes **1**–**3** to a periodic sheet of graphene (**Figure** **4**). For **Ir‐1**, **Ir‐2,** and **Ir‐3**, we calculate binding energies of −30.3, −22.1, and −31.4 kcal mol^−1^, respectively. **Ir‐1** and **Ir‐3** have binding energies within 1.2 kcal mol^−1^ of each other, both of which are significantly larger than the binding energy for **Ir‐2**. In **Ir‐1** and **Ir‐3**, the pyrenyl ligand group binds parallel to the graphene surface, suggesting favorable π‐stacking of the molecular complexes with the carbon support (Figure 4). Here, the angles made between the graphene and the phenyl groups of **Ir‐2** are 42.0, 46.4, and 58.7°.

**Figure 5:**
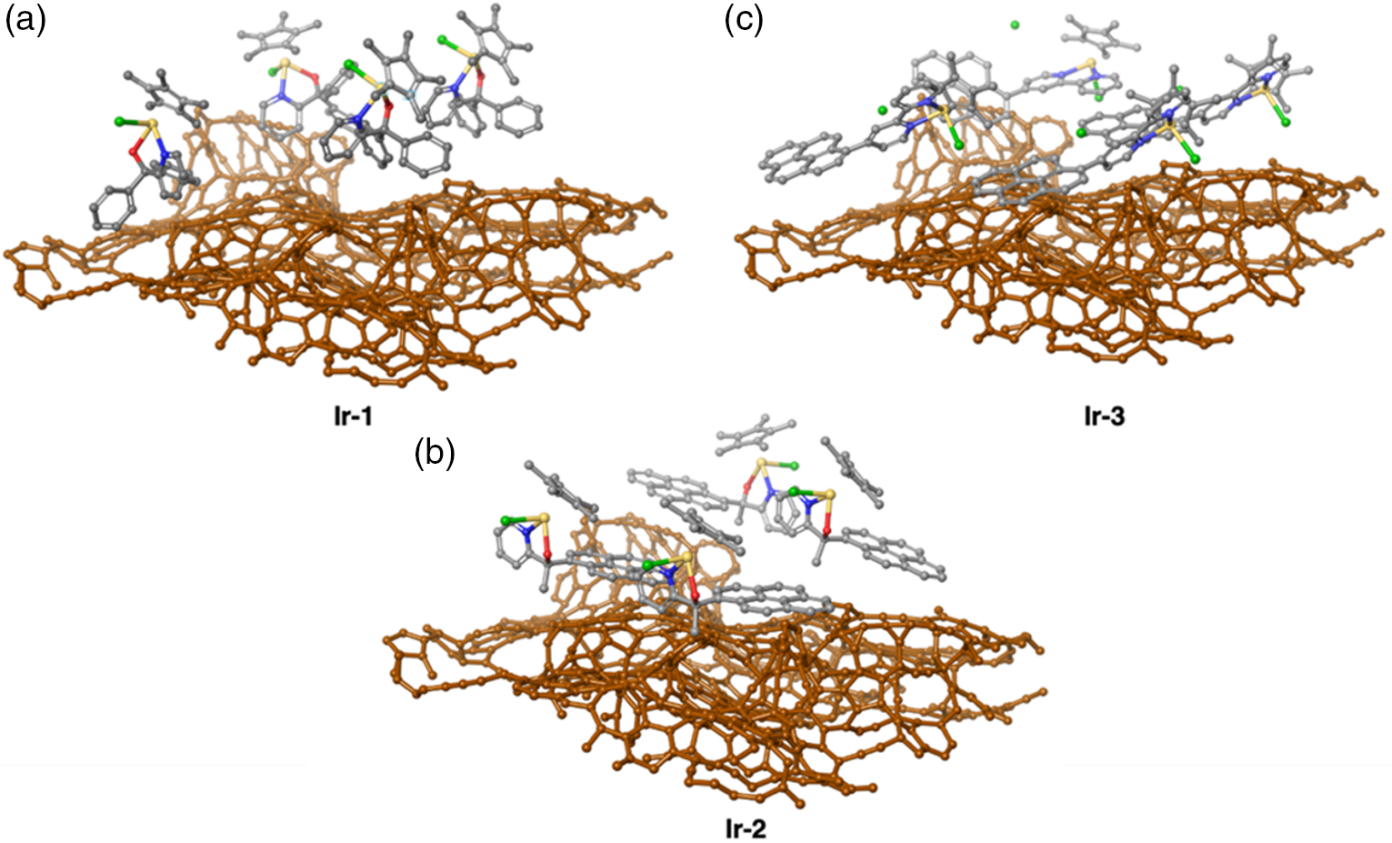
UFF‐predicted binding for four of each Ir complex (numbered) on the LDAC surface (brown atoms).^[^ ^56^ ^]^ Given the variation of the carbon surface, four molecules were used to sample the different local topologies present. The average per molecule binding energy was calculated simply by dividing the total system binding energy by 4. Hydrogen atoms omitted for clarity.

We also probed the binding of the Ir structures to a LDAC surface with UFF (**Figure** **5**).^[^ ^56^ ^]^ Given the significant variation of the carbon surface's topology, four identical Ir complexes were bound to the support in each simulation, and the binding energy was averaged over the four molecules. The average per‐molecule binding energies for **Ir‐1**, **Ir‐2,** and **Ir‐3** to the LDAC surface were calculated as −32.4, −27.5, and −30.6 kcal mol^−1^. The binding energy for **Ir‐2** to the LDAC surface increased dramatically (−5.4 kcal mol^−1^) compared with the analogous binding to graphene. **Ir‐1** saw an improved binding energy of −1.9 kcal mol^−1^ to the amorphous surface, while **Ir‐3**'s binding energy actually decreased 0.8 kcal mol^−1^ relative to its binding to graphene. Consequently, we find that **Ir‐1** binds the strongest to the amorphous surface followed by **Ir‐3**; the different trend was observed for graphene binding. From the energy evaluation, **Ir‐2** presents the lowest binding energy on both graphene and LDAC surfaces, and its difference with **Ir‐1** and **Ir‐2** is smaller on LDAC.

As quantification of catalytic active sites is necessary to evaluate intrinsic activity of an electrocatalyst, we must contextualize the binding energy with respect to the surface area necessary to host each Ir complex to explain the experimental loading trend. Here, we focus on Ir complexes binding to graphene as its flat topology makes it easier to deduce surface area coverage. For quantification, the surface “footprint” is defined as the number of graphene carbon atoms required to bind the Ir complex. For **Ir‐1**, the footprint consists of 35 atoms, meaning that complex **1** requires 35 carbon atoms of the graphene sheet to achieve optimal π‐stacking. The footprint for **Ir‐3** is 54 atoms, which is nearly 54% larger than the footprint for **Ir‐1** (**Table** **1**). By visual inspection, it is clear that the footprint for **Ir‐2** is significantly smaller than that of **Ir‐1** and **Ir‐3**, due to the absence of the large pyrenyl moiety in **Ir‐1** and **Ir‐3**. The footprint for optimal binding of **Ir‐2** is only 23 atoms. Overall, **Ir‐2** requires the smallest area on the carbon support, followed by **Ir‐1** then **Ir‐3**. With these footprints defined, we calculate the binding energy per unit surface area (which we define as C^−1^ for per carbon atom in footprint): −0.87 kcal mol^−1^ C^−1^ for **Ir‐1**, −0.96 kcal mol^−1^ C^−1^ for **Ir‐2**, and −0.58 kcal mol^−1^ C^−1^ for **Ir‐3**. Note that these values are for binding to graphene. Using the same footprints, we also evaluate binding energies per unit area for the LDAC surface: −0.93, −1.20, and −0.57 kcal mol^−1^ C^−1^ for **Ir‐1**, **Ir‐2**, and **Ir‐3** respectively. This descriptor best reflects the experimentally observed complex loading efficiency for **Ir 1‐3** because it contains information about the binding energy and the surface coverage on a realistic carbon support. **Ir‐2** has the highest binding energy per footprint atom at −1.20 kcal mol^−1^ C^−1^, followed by **Ir‐1** at −0.93 kcal mol^−1^ C^−1^, then finally **Ir‐3** with −0.57 kcal mol^−1^ C^−1^. With the lowest footprint and highest binding energy per footprint, **Ir‐2** exhibits the highest complex loading efficiency (2.95% Ir mass loading according to ICP).^[^ ^62^ ^]^

**Table 1:** Footprints and Binding Energies of Ir Complexes **1‐3** on Graphene and Low‐Density Amorphous Carbon

Ir Complex	Footprint (# atoms)	Binding Energy to Graphenea)	Graphene binding per area^b^	Binding Energy to LDACa)	LDAC binding per areab)
**Ir‐1**	35	−30.3	−0.87	−32.4	−0.93
**Ir‐2**	23	−22.1	−0.96	−27.5	−1.20
**Ir‐3**	54	−21.4	−0.58	−30.6	−0.57

a) kcal mol^−1^;

b) kcal mol^−1^ C^−1^.

### 2.4 Electrocatalytic Water Oxidation Using Ir–Tethered OMCs

The OMCs modified by Ir complexes retain the ordered porous structure (Figure 3b and Figure S9, Supporting Information) and were studied for the OER catalysis (see Experimental Section). The OER catalytic analyses were carried out in a O_2_‐saturated 0.5 m H_2_SO_4_ aqueous solution. LSV curves from 0.3 to 1.63 V versus RHE at a scan rate of 10 mV s^−1^ in Figure 3c show that **Ir‐2** delivers a higher current density than **Ir‐1** and **Ir‐3** at the same overpotential. For example, at overpotential of 300 mV, **Ir‐2** delivered a current density of 8.8 mA cm^−2^, larger than 7.0 and 4.9 mA cm^−2^ for **Ir‐1** and **Ir‐3**, respectively. Further, the overpotential of **Ir‐2** at a current density of 10 mA cm^−2^ is 320 mV, which is lower than **Ir‐1** (364 mV) and **Ir‐3** (385 mV). The oxidation peaks at 0.58 V and 0.65 V for **Ir‐1** and **Ir‐3** in LSV plot (Figure 3c) were assigned to oxidation of the pyrene moiety in **L1** and **L3**.^[^ ^46^, ^63^, ^64^ ^]^ To validate the OER activity originates from Ir, OMC loaded with metal‐free ligands (pyrene‐pyalk **L1** and diphenyl derivative **L2**) were also tested under the same conditions (Figure S10, Supporting Information). It was observed that the current densities from ligand or OMC were much smaller than I R‐tether OMCs.

The OER TOFs of Ir‐tethered OMCs were calculated to evaluate the intrinsic activity of each Ir site (**Table** **2**). The three catalysts exhibited similar TOFs, with **Ir‐2** delivering a slightly lower TOF than **Ir‐1** and **Ir‐3**. For example, at 300 mV overpotential, **Ir‐2** presents a TOF of 0.9 s^−1^ per Ir, which is lower than **Ir‐1** (1.3 s^−1^) and **Ir‐3** (1.6 s^−1^). However, due to more efficient immobilization of **Ir‐2** on OMC as discussed earlier, **Ir‐2** allows the incorporation of more catalyst sites on the OMC surface and thus displays the highest OER current density among three catalysts. These TOF values are comparable with other supported Ir catalysts, and more importantly, ours operate at lower overpotentials and pH (Table 2),^[^ ^35^, ^36^, ^37^, ^38^, ^41^ ^]^ which is desirable for the improved device energy efficiency.

**Table 2:** TOFs of **Ir‐1,** **Ir‐2,** and **Ir‐3** in Comparison with Reported Examples

Entry	Catalyst	pH	*η* (mV)	TOF ([s]^−1^)	Reference
1	[Cp*Ir(bpy)Cl]@glassy carbon	5.0	660	1.7a)	35
2	[Cp*Ir(2‐phenylpyridine)Cl]@glassy carbon	5.0	660	3.3a)	35
3	[Ir(cod){MeIm(CH_2_)_3_SO_3_}]@carbon nanotubes	7.0	790	6.1a)	36
4	[Cp*Ir(PO_3_H_2_‐bpy)(OH_2_)]@indium tin oxide	4.0	750	6.7b)	38
5	[Ir(pyalk)(OH_2_)_2_(μ—O)]_2_@indium tin oxide	2.6	520	7.9c)	41
6	**Ir‐1**	0.3	200/300/400	0.7/1.3/2.5a)	This work
7	**Ir‐2**	0.3	200/300/400	0.5/0.9/1.8a)	This work
8	**Ir‐3**	0.3	200/300/400	0.8/1.6/3.7a)	This work

Bpy = 2,2′‐bipyridine; pyalk = 2‐(2′‐pyridyl)‐2‐propanolate; MeIm = 3‐(propyl‐3‐sulfonate)‐imidazol‐2‐ylidene.

a) TOF determined from the catalytic current from LSV and loading from ICP measurements.

b) TOF determined from current density in controlled potential electrolysis.

c) TOF determined from O_2_ measurements and Ir loadings were determined from integration of the Ir^III^/Ir^IV^ redox wave.

**Figure 6:**
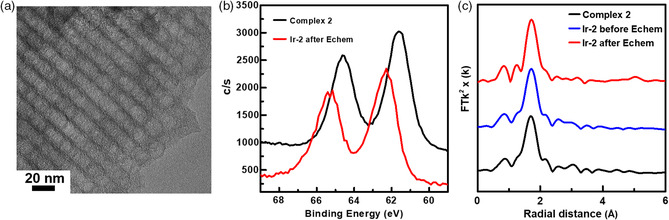
a) TEM image of **Ir‐2** after stability tests of 1000 LSV scans (0.3–1.63 V versus RHE); b) XPS spectra of complex **2** and **Ir‐2** after electrochemical conditions; c) EXAFS analysis of complex **2** and **Ir‐2** for before and after electrochemical conditions.

The stabilities of Ir‐tethered OMC catalysts were studied using continuous LSV scans. Among the three catalysts, **Ir‐2** exhibited the best stability after 1000 LSV scans from 0.3 to 1.65 V versus RHE (Figure 3d and Figure S11, Supporting Information). Furthermore, a TEM image of **Ir‐2** (**Figure** **6**a and Figure S12, Supporting Information) after continuous 1000 LSV scans shows that the OMC structure was well preserved without the formation of visible IrO_2_ nanoparticles.

We used a suite of spectroscopic probes to elucidate the electronic structures and atomic coordination of Ir on OMC before and after OER stability tests. Figure 6b and Figure S13, Supporting Information, shows the Ir 4f spectra of Ir complexes and their heterogenized catalysts measured by X‐ray photoelectron spectroscopy (XPS). Prior to the OER test, the Ir complex **2** exhibited Ir 4f_7/2_ peaks in the range of 61.6–62.4 eV, suggesting a primary Ir(III) oxidation state in these materials, which agrees with reported complexes [Cp*Ir(2‐2′‐pyridyl)‐2‐propanolate)Cl]^[^ ^26^ ^]^ and [Cp*Ir(1‐(4‐(*tert*‐butyl)pyridin‐2‐yl)‐3‐methyl‐1 H‐imidazol‐3‐ium iodide)Cl]Cl.^[^ ^65^ ^]^ The Ir 4f_7/2_ peaks were shifted to higher binding energy 62.7 eV after the OER test, which indicates the Ir evolution to Ir^4+^ oxidation state under the OER potentials. Ir L‐edge extended X‐ray absorption fine structures (EXAFSs), obtained from synchrotron radiation XAS, were also monitored to reveal the Ir single‐site structural change (Figure 6c and Figure S14–S23, Supporting Information). It was clearly seen that all Ir/OMC catalysts present almost unchanged Ir Fourier‐transformed EXAFS spectra before and after the OER stability test, which are also consistent with pristine as‐synthesized Ir complexes powders. For **Ir‐2** sample suite (Figure 6c), only two peaks at 1.84 and 2.07 Å were observed, which is associated with Ir—O and Ir—N scattering pathways in the complex. These peaks are distinct to typical Ir—O scattering pathway in Ir oxide materials (1.94–1.98 Å).^[^ ^66^ ^]^ More importantly, the representative Ir—Ir scattering pathway in the range of 2.70–3.83 Å for Ir metal or metal oxides became undetectable in our samples,^[^ ^67^ ^]^ which is consistent with the Ir complexes immobilized on OMC in **Ir‐1**, **Ir‐2,** and **Ir3** being robust against aggregation and sintering to clusters/nanoparticles in the OER catalysis.

## 3 Conclusions

Rational design of the structure of molecular catalysts is demonstrated as a methodological advance for integrating homogenous catalyst on heterogeneous support interface. Well‐defined Ir catalytic centers were designed and synthesized from Ir molecular complexes. The successful immobilization of Ir molecular catalysts on the surface of OMC materials was achieved via noncovalent π‐stacking interactions for OER. The resultant hybrid electrodes exhibit an increase in the stability of the complexes in acidic environment and preserve the Ir single‐site structure under OER conditions, as indicated using XAS investigation. Theoretical DFT calculations, which are validated by the experimental results, provide vivid understanding of immobilization of molecular catalysts on carbon support materials that will aid in the future rational integration of high‐performance homogeneous electrocatalysts into heterogeneous systems.

## 4 Experimental Section

4.1

4.1.1

##### Chemicals and Materials

FeCl_3_.6H_2_O (98%), oleic acid (OAc, 90%), and 1‐octadecene (ODE, 90%) were bought from Sigma Aldrich. Hexane (ACS Certified), hydrochloric acid (HCl), ethanol (200 proof), 2‐propanol (IPA, ACS Certified), and KOH (ACS Certified) were purchased from Fisher Scientific. Sodium oleate was purchased from Tokyo Chemical Industry. Literature procedures were used to prepare 4‐(1‐pyrenyl)‐2,2′‐bipyridine,^[^ ^58^ ^]^ [Cp*Ir(μ—Cl)_2_]_2_,^[^ ^68^ ^]^ and Cp*Ir{diphenyl(2‐pyridyl)methanolate‐κO,κN}Cl (**2**).^[^ ^57^ ^]^

##### General Methods

All solvents were reagent grade or better. Deuterated solvents were used as received. All the solvents were kept in the glovebox over 4 Å molecular sieves. All NMR spectra were recorded on a Varian Inova 600 or 500 MHz spectrometer or a Bruker Avance III 800 MHz spectrometer. The operating frequency for ^13^C{^1^H} NMR was 150 MHz (on the 600 MHz instrument) or 201 MHz (on the 800 MHz instrument). All ^1^H and ^13^C{^1^H} NMR spectra were referenced against residual ^1^H resonances (^1^H NMR) or ^13^C{^1^H} resonances (^13^C{^1^H} NMR) of the deuterated solvents. All spectra were recorded at 25 °C unless otherwise indicated.

##### Synthesis of 1–Pyrenyl(2‐Pyridyl)ethanol (**L1**)

Degassed 2‐bromo pyridine (12.7 mmol) was dissolved in dry tetrahydrofuran (THF) (40 mL) and the solution cooled to −78 °C. *n*–BuLi (2.5 m in hexanes, 14 mmol) was added via cannula over 20 min observing a color change to red. The resulting solution was stirred for 3 h at −78 °C observing a color change to brown. Then, a solution of acetylpyrene (12.7 mmol) in dry THF (10 mL) was added via cannula over 10 min. The solution was left to warm to room temperature and stirred for 18 h, after which it had turned dark green. The reaction was quenched by addition of 1 M NaOH (25 mL) and water (25 mL), changing to yellow. The product was extracted with diethyl ether (3 × 50 mL), the combined organic extracts dried over MgSO_4_, filtered and dried in vacuo to afford the crude product as a pale‐yellow powder. The powder was washed with toluene (2 × 20 mL) to yield a white solid (1.2 g, 27%). ^1^H NMR (600 MHz, CDCl_3_): *δ* = 8.70 (dt, *J* = 5.0, 1.4 Hz, 1 H, Ar‐pyridine), 8.37 (d, *J* = 8.0 Hz, 1 H, Ar), 8.21 (d, *J* = 8.0 Hz, 1 H, Ar), 8.18 (dd, *J* = 9.4, 1.6 Hz, 1 H, Ar), 8.14 (dd, *J* = 7.6, 1.1 Hz, 1 H, HAr), 8.10–8.03 (m, 3 H, HAr), 7.95 (t, *J* = 7.6 Hz, 1 H, Ar), 7.82 (d, *J* = 9.4 Hz, 1 H, Ar), 7.43 (tt, *J* = 7.7, 2.0 Hz, 1 H, Ar‐pyridine), 7.19 (td, *J* = 5.0, 2.4 Hz, 1H, Ar‐pyridine), 6.79 (dt, *J* = 7.9, 1.1 Hz, 1 H, Ar–Pyridine), 5.96 (br s, 1H, OH), 2.21 (s, 3H, CH_3_). ^13^C{^1^H} NMR (151 MHz, CDCl_3_) δ 166.7, 147.5, 138.1, 137.2, 131.6, 131.3, 130.4, 129.6, 127.7, 127.4, 126.7, 126.2, 126.1, 126.0, 125.2, 125.2, 125.1, 124.8, 124.2, 122.1, 120.8, 32.7.

##### Synthesis of Cp*Ir(1‐Pyrenyl(2‐Pyridyl)ethanolate‐κO,κN)Cl (**1**)

[Cp*Ir(μ–Cl)_2_]_2_ (70.0 mg, 0.088 mmol), 2‐pyrenyl(2‐pyridyl)ethanol (59.6 mg, 0.18 mmol, 2.1 equivalents) and Na_2_CO_3_ (65.0 mg, 0.61 mmol) were dissolved in an acetone/CH_2_Cl_2_ mixture (15 mL:10 mL). The resulting orange solution was stirred for 16 h at 50 °C, after which time the solution had turned yellow. MgSO_4_ was added and the solution was filtered and the solvent removed in vacuo to afford a yellow solid. The yellow solid washed with toluene (2 × 5 mL) and the powder dried in vacuo (114.5 mg, 95%). Single crystals for X‐ray diffraction were obtained from slow evaporation of a chloroform solution. ^1^H NMR (800 MHz, CDCl_3_): δ = 10.23 (br s, 1 H, Ar), 8.73 (d, *J* = 5.6 Hz, 1 H, Ar‐pyridine), 8.20 (br s, 2 H, Ar), 8.14 (d, *J* = 7.4 Hz, 1 H, Ar‐pyridine), 8.10 (br s, 1H, Ar), 8.01 (d, *J* = 8.7 Hz, 1H, Ar‐pyridine), 7.95 (d, *J* = 8.8 Hz, 1 H, Ar‐pyridine), 7.92–7.77 (m, 2H, Ar), 7.48 (s, 1H, Ar), 7.37 (s, 1H, Ar), 6.87 (br s, 1H, Ar), 2.32 (s, 3H, CH_3_), 1.17 (s, 15H, Cp*). Elemental analysis C_33_H_31_ClIrNO (685.284): calcd. C 57.84 H 4.56 N 2.04; found C 57.67, H 4.55 N 2.05.

##### Synthesis of [Cp*Ir(4‐(1‐Pyrenyl)‐2,2‐Bipyridine)Cl]Cl′ (**3**)

[Cp*Ir(μ–Cl)_2_]_2_ (70.0 mg, 0.088 mmol) and pyrene‐bpy (59.6 mg, 0.18 mmol, 2.1 equivalents) were suspended in CH_2_Cl_2_ (10 mL) and stirred at room temperature for 16 h. The solvent was removed and the yellow solid was washed with Et_2_O (3 × 3 mL). The solid was dried under vacuum (86.7 mg, 71%). Single crystals for X‐ray diffraction were obtained from slow evaporation of a chloroform solution. ^1^H NMR (600 MHz, CDCl_3_): δ = 8.99 (dd, *J* = 5.7, 2.8 Hz, 1H, Ar‐pyridine), 8.87 (d, *J* = 5.6 Hz, 1H, Ar‐pyridine), 8.73 (s, 1H, Ar), 8.65 (d, *J* = 8.0 Hz, 1H, Ar), 8.23–8.20 (m, 1H, Ar), 8.16 (t, *J* = 7.2 Hz, 2H, Ar), 8.12 (t, *J* = 7.1 Hz, 2H, Ar), 8.08–8.03 (m, 3H, Ar), 8.02–7.99 (m, 1H, Ar), 7.97 (t, *J* = 7.6 Hz, 1H, Ar), 7.85–7.81 (t, *J* = 6.6 Hz, 1H, Ar‐pyridine), 1.67 (s, 15H, Cp*).^13^C{^1^H} NMR (201 MHz, CDCl_3_): δ = 155.8 (bpy), 155.4 (bpy), 153.6 (bpy), 151.6 (bpy), 150.9 (bpy), 140.8 (bpy), 132.7, 131.4, 131.1, 130.8, 130.5, 129.6 (bpy), 129.4, 129.1, 128.2, 128.0, 127.4, 126.7, 126.4, 126.1, 126.0 (bpy), 125.5 (bpy), 125.3, 125.0, 124.7, 123.3, 89.7 (Cp*), 9.2 (Cp*). Elemental analysis C_36_H_31_Cl_2_IrN_2_ (754.775): calcd. C 57.29 H 4.14 N 3.71; found C 57.83, H 4.32 N 3.45.

##### Synthesis of Fe_3_O_4_ Nanoparticles

Monodisperse Fe_3_O_4_ nanoparticles were synthesized with some modifications from previous reported work.^[^ ^51^ ^]^ Briefly, iron oleate was prepared as a precursor by refluxing the mixture of FeCl_3_.6H_2_O, sodium oleate, hexane, ethanol, and deionized (DI) water at 50 °C for 4 h. The Fe_3_O_4_ synthesis was typical colloidal synthesis with moisture free condition. The mixture of iron oleate (3.2 g), ODE (20 mL), and oleic acid (0.64 mL) was degassed under vacuum at 100 °C, then heated to 310 °C in N_2_, and subsequently kept at the temperature for 1 h. After removal of the heating mantle and cooling down to room temperature, the product was precipitated with IPA and separated with centrifugation at spin speed of 8000 rpm for 8 min. The procedure was repeated twice. The obtained Fe_3_O_4_ nanoparticles were dispersed in hexane for further use.

##### OMC Preparation

The OMCs were prepared by a strategy using monodisperse Fe_3_O_4_ nanoparticle superlattices as a sacrificial template. The colloidal dispersion of Fe_3_O_4_ nanoparticles was slowly dried in ambient condition to obtain the self‐assembled superlattice. To carbonize the oleic acid surfactant capping the Fe_3_O_4_ nanoparticles, the obtained superlattice was annealed in N_2_ under 500 °C for 2 h. Concentrated HCl was used for the removal of Fe_3_O_4_ template to yield OMC. The OMC material was further annealed in forming gas at 900 °C for graphitizing process.

##### Ir–Loaded OMC Preparation

The OMC and Ir complex (weight ratio 1/1) were mixed and dispersed in isopropanol for sonication. The complex‐loaded OMC was separated by centrifugation, and the supernatant solution containing untethered complexes was removed. The obtained complex‐loaded OMC was further sonicated in IPA. After precipitating for 0.5 h, the supernatant solution with free Ir complexes was removed for UV–vis analysis. The precipitant was dried and used for further ink preparation.

##### Electrocatalytic OER Measurement

All electrocatalytic performance was characterized at room temperature in the O_2_‐saturated 0.5 m H_2_SO_4_ aqueous electrolyte. The three‐electrode testing cell consisted of a glassy carbon working electrode, a Pt foil counter electrode, and a Ag/AgCl (3.0 m KCl) reference electrode. The tests were conducted with a BioLogic (Model VMP3) potential station. The electrode ink was prepared by sonicating Ir‐loaded OMC, IPA, and nafion solution. The volume ratio of nafion/IPA is 1/100. The concentration of Ir‐loaded OMC in the ink is 5 mg mL^−1^. The working electrode was fabricated by spin coating 20 μL ink on polished glassy carbon. All the potentials were reported versus RHE using the equation: *E*(vs RHE) = *E*(vs Ag/AgCl) + 0.220 V, where 0.220 V is the potential difference between the Ag/AgCl (3 m KCl) reference electrode and RHE in 0.5 m H_2_SO_4_ that is calibrated via open circuit voltage test prior to the electrocatalysis. The overpotential (*η*) for OER could be calculated using *η *= *E* (vs RHE) −1.23 V. The OER activity was examined by linear sweep voltammogram (LSV) from 0.3 to 1.63 V versus RHE at a scan rate of 10 mV s^−1^. The stability of the catalysts was evaluated by continuous LSV scans at a scan rate of 10 mV s^−1^. TOF is calculated by TOF = $\frac{j_{\text{Geo}}\times s}{4\times \;N_{\text{Ir}-\text{atom}}\times \;F}$, where *j*_Geo_ is geometric current density on LSV plot, *s* is geometric area of the work electrode, *N*_Ir‐atom_ is the amount of Ir atoms calculated from loading amount, and *F* is the Faraday constant.

##### Material Characterization

TEM images were taken on FEI Spirit (120 kV). Loading amounts of Ir on OMC were obtained with ICP‐OES on a PerkinElmer Avio‐200 ICP spectrometer. Ir‐loaded OMC was boiled in aqua regia for 1 h within an encapsulated vial at 120 °C. The obtained solution was diluted with 2% nitric acid aqueous solution and further used for a measurement of Ir concentration. XPS was carried out using PHI VersaProbe III that was equipped with monochromatic Al Kα X‐rays (1486.6 eV) and spherical capacitor energy analyzer to identify the surface composition and electronic structure differences before and after electrochemistry. Spectra were measured with a 100 μm spot size and with a 69 eV pass energy. Data were analyzed in PHI Multipak 9.8.0.19, where a Shirley background was subtracted to remove the inelastic component. The binding energy scale was charge referenced to the C 1s peak of the supporting graphitic carbon at 284.5 eV. The ex situ Ir L‐edge EXAFS spectra were collected at room temperature in the fluorescence mode at the beamline 20BM of Advanced Photon Source, at the Argonne National Laboratory. The processing of EXAFS raw data was carried out by the standard procedure with ATHENA program.^[^ ^69^ ^]^ The least‐squares curve fitting analysis of the EXAFS *χ*(*k*) data was processed by the ARTEMIS program. The model was built based on single crystal information from X‐ray diffraction. The function *F*(*k*), *λ* (the photoelectron mean free path for all paths in Å) and *ϕ*(*k*) (phase shifts) were calculated by the ab initio code FEFF 9.05. Raman measurements were carried out on a Renishaw InVia confocal Raman microscope with an Ar^+^ excitation laser wavelength of 514 nm. Spectra were recorded from 0 to 1900 cm^−1^ with 30 s integration times under a 50× objective lens. The reported spectrum was the cumulative addition of four measurements.

##### Singe–Crystal X–Ray Diffraction Details

A suitable single crystal of **1** or **3** was coated with paratone oil and mounted on a MiTeGen MicroLoop. The X‐ray intensity data were measured on a Bruker Kappa APEXII Duo system equipped with a fine‐focus sealed tube (Mo Kα, *λ* = 0.71073 Å) and a graphite monochromator. The frames were integrated with the Bruker SAINT software package^[^ ^70^ ^]^ using a narrow‐frame algorithm. Data were corrected for absorption effects using the multiscan method (SADABS).^[^ ^69^ ^]^ Each structure was solved and refined using the Bruker SHELXTL Software Package^[^ ^71^ ^]^ within APEX3^[^ ^69^ ^]^ and OLEX2.^[^ ^72^ ^]^ Nonhydrogen atoms were refined anisotropically. Hydrogen atoms were placed in geometrically calculated positions with *U*_iso_ = 1.2*U*_equiv_ of the parent atom (*U*_iso_ = 1.5*U*_equiv_ for methyl). For **3**, the Cp* ligand and the Ir atom were each disordered over two positions. The relative occupancies were freely refined, and constraints were used on the anisotropic displacement parameters of the disordered ligand.

##### Computational Methods

Finite DFT calculations were carried out using the Jaguar v10.9 software by Schrodinger Inc. Geometry optimizations were carried out using the B3LYP hybrid functional with the Grimmie–Becke–Johnson D3 correction for London Dispersion interactions. The 6–31 G*+ basis set was used for organics, whereas the Ir was described with the Los Alamos large‐core pseudopotential with triple‐zeta quality, augmented with polarization and diffuse functions (designated LAV3P*+ in Jaguar). Periodic DFT calculations were carried out using VASP v5.4.4. The PBE generalized gradient approximation functional (including the D3 correction for London Dispersion forces) was used for optimizations. The plane wave cutoff was set to 500 eV, and a 1 × 1 × 1 gamma‐centered K‐point grid was used. Pseudopotentials based on the projector augmented wave method were used for all atoms. Classical dynamics (used to model complex binding to the low‐density carbon surface) based on the reactive force field were simulated using LAMMPS. Carbon–Carbon van der Waals (VDW) interactions were fitted to match the VDW distance and energy of two stacked graphene sheets. More details are included in Calculation Details section, Supporting Information.

## Conflict of Interest

The authors declare no conflict of interest.

## Data Availability Statement

The data that support the findings of this study are available from the corresponding author upon reasonable request.

## Supporting information

Supplementary Material
