# Supplementary material for: Noncovalent Immobilization of Pentamethylcyclopentadienyl Iridium Complexes on Ordered Mesoporous Carbon for Electrocatalytic Water Oxidation
Source: Small Sci. 2021 Aug 6;1(11):2100037. doi: 10.1002/smsc.202100037 (PMC11935816; doi:10.1002/smsc.202100037)
Supplement: Supplementary file 1 — Supplementary Material [file SMSC-1-2100037-s001.pdf]

Supporting Information

for

Non-covalent Immobilization of Pentamethylcyclopentadienyl Iridium Complexes on Ordered Mesoporous Carbon for Electrocatalytic Water Oxidation

Ana M. Geer[†,‖], Chang Liu[†,‖], Charles Musgrave[‡], Christopher Webber[†], Grayson Johnson[†], Hua Zhou[#], Cheng-Jun Sun[#], Diane A. Dickie[†], William A. Goddard III[‡]*, Sen Zhang[†]*, T. Brent Gunnoe[†]*

[†]Department of Chemistry, University of Virginia, Charlottesville, VA 22904

[‡]Materials and Process Simulation Center, Department of Chemistry, California Institute of Technology, Pasadena, CA 91125

[#] Advanced Photon Source, Argonne National Laboratory, Lemont, Illinois 60439, USA

[‖]A.M.G. and C.L. contributed equally.

Crystallographic Section

Table S1: Crystallographic data for **1** and **3**.

	**1**	**3**
**CCDC**	2069449	2069450
**Chemical formula**	$C_{36}H_{34}Cl_{10}IrNO$	$C_{39}H_{34}Cl_{11}IrN_2$
**FW (g/mol)**	1043.34	1112.83
**T (K)**	100(2)	100(2)
**λ (Å)**	0.71073	0.71073
**Crystal size (mm)**	0.110 x 0.260 x 0.626	0.093 x 0.127 x 0.161
**Crystal habit**	orange plate	yellow block
**Crystal system**	monoclinic	monoclinic
**Space group**	$P\ 2_1/c$	$P\ 2_1/n$
**a (Å)**	17.5197(12)	9.1820(11)
**b (Å)**	10.3992(7)	20.112(2)
**c (Å)**	22.8431(15)	22.822(3)
**α (°)**	90	90
**β (°)**	108.982(2)	93.185(4)
**γ (°)**	90	90
**V ($Å^3$)**	3935.5(5)	4208.0(9)
**Z**	4	4
**ρ_{calc} ($g/cm^{3)}$**	1.761	1.757
**μ (mm^{-1})**	4.103	3.904
**F(000)**	2048	2184
**θ range (°)**	1.23 to 29.61	1.35 to 25.44
**Index ranges**	$-24 \leq h \leq 24$<br>$-14 \leq k \leq 14$<br>$-31 \leq l \leq 31$	$-11 \leq h \leq 11$<br>$-23 \leq k \leq 24$<br>$-27 \leq l \leq 27$
**Reflns coll.**	54344	37567
**Ind. reflns**	11057 [R_{int} = 0.0301]	7750 [R_{int} = 0.1004]
**Data / restraints / parameters**	11057 / 0 / 436	7750 / 0 / 471
**Goodness-of-fit on F^2**	1.059	1.037
**R_1 [I>2σ(I)]**	0.0254	0.0603
**wR_2 [all data]**	0.0526	0.1839

Experimental Section

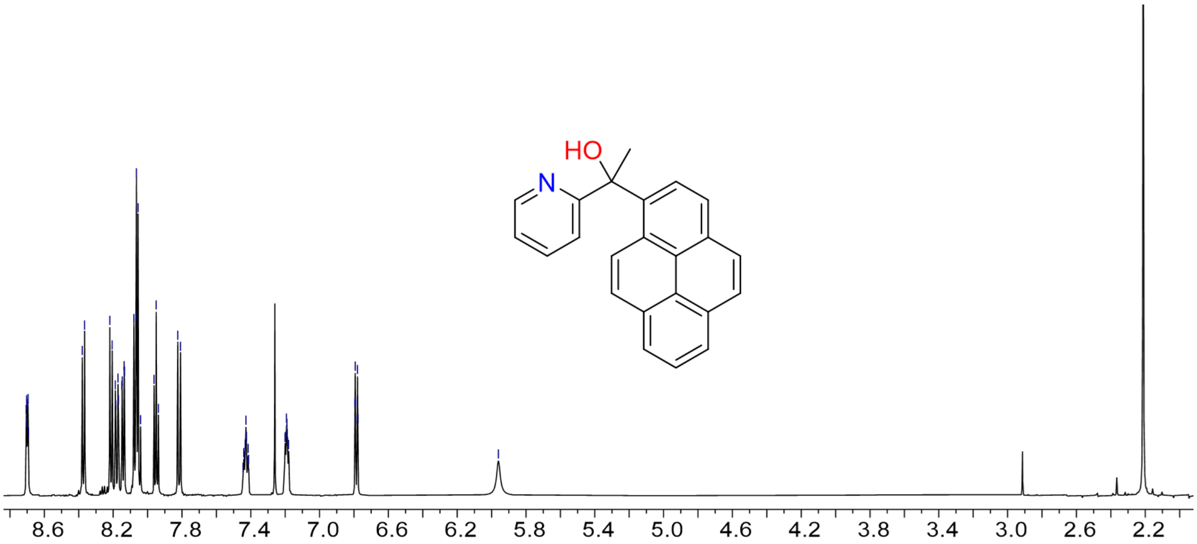

Figure S1. ^{1}H NMR spectrum of 1-pyrenyl(2-pyridyl)ethanol (**L1**).

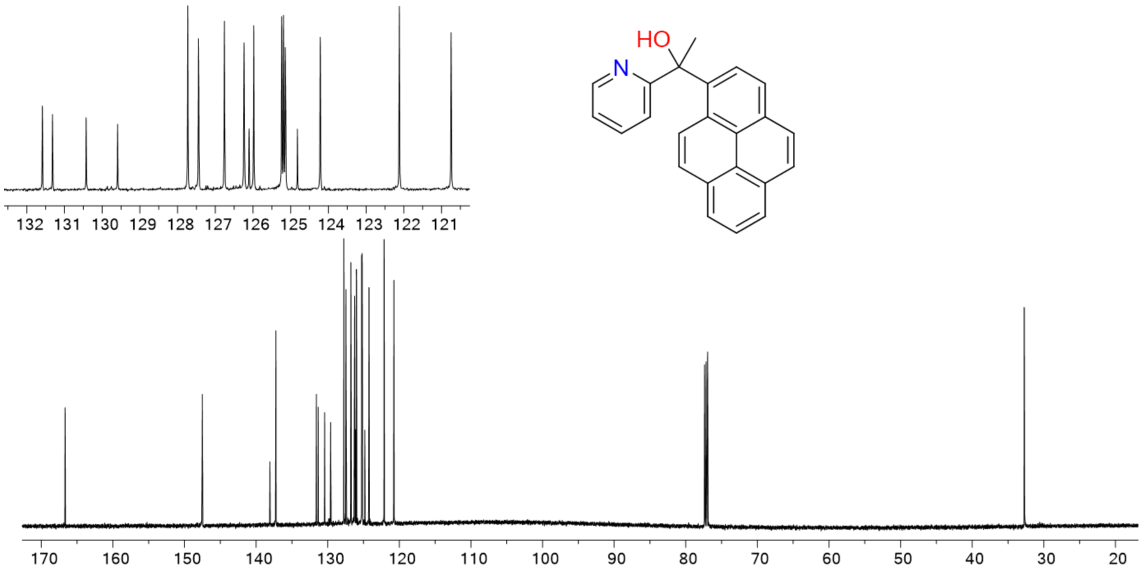

Figure S2. ^{13}C{^{1}H} NMR spectrum of 1-pyrenyl(2-pyridyl)ethanol (**L1**).

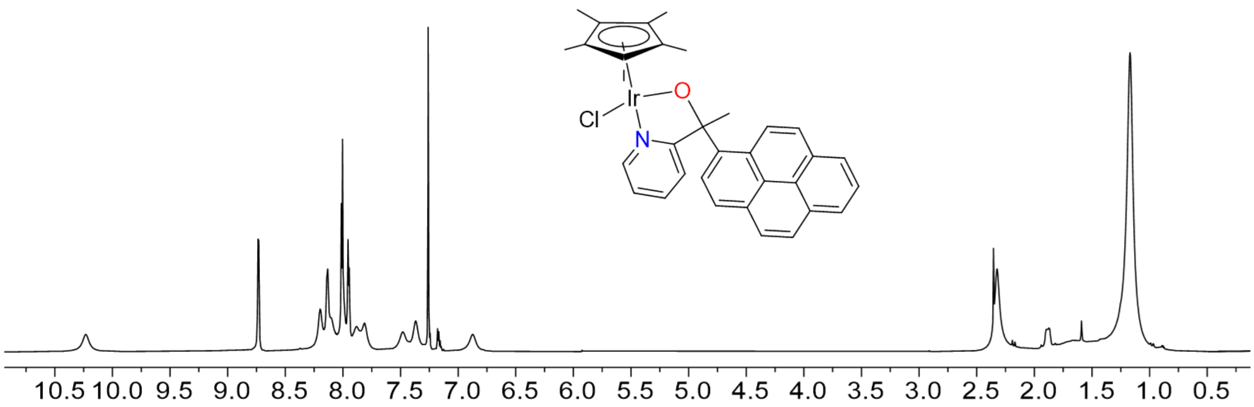

Figure S3. ^{1}H NMR spectrum of Cp*Ir(1-pyrenyl(2-pyridyl)ethanolate-κO,κN)Cl (**1**).

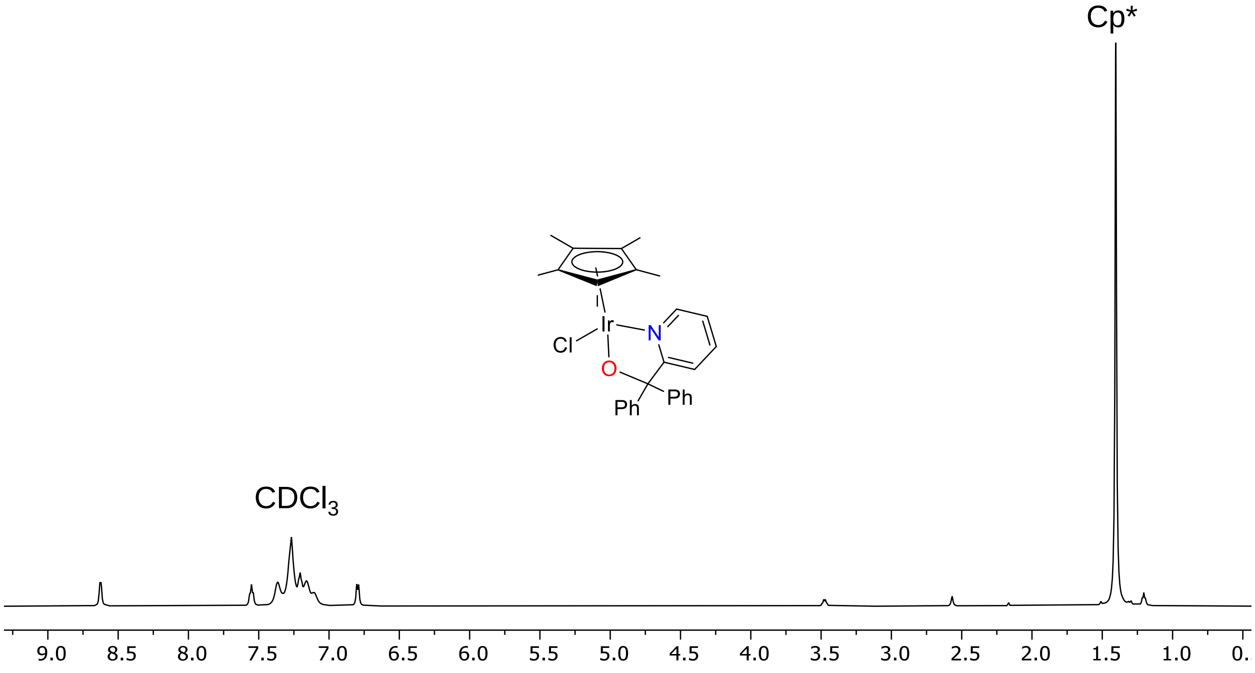

Figure S4. ^{1}H NMR spectrum of Cp*Ir{diphenyl(2-pyridyl)methanolate-κO,κN}Cl (**2**).

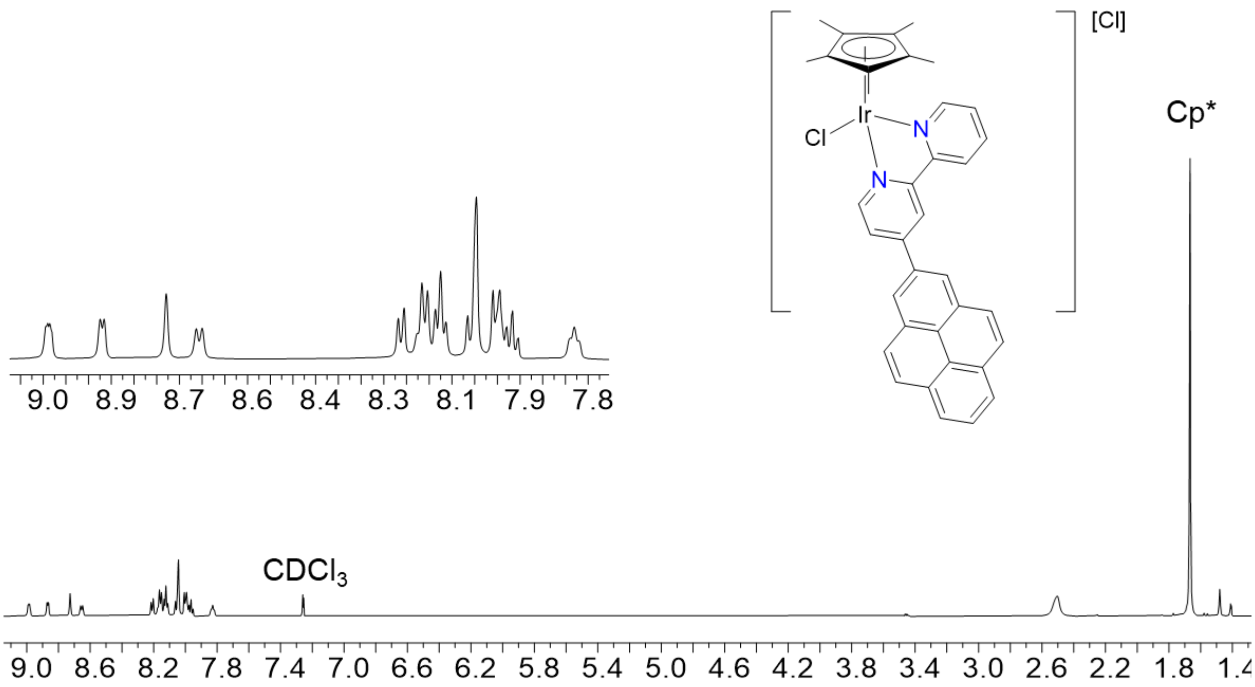

Figure S5. ^{1}H NMR spectrum of [Cp*Ir(4-(1-pyrenyl)-2,2'-bipyridine)Cl]Cl (**3**).

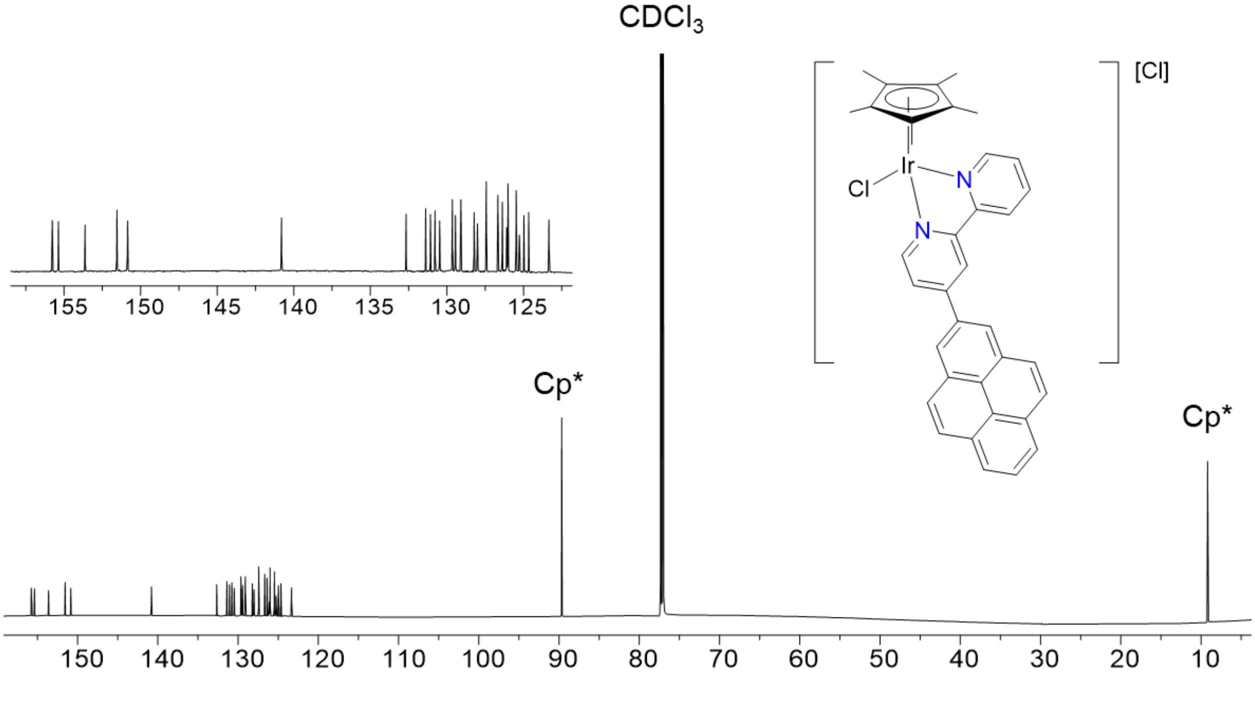

Figure S6. ^{13}C{^{1}H} NMR spectrum of [Cp*Ir(4-(1-pyrenyl)-2,2'-bipyridine)Cl]Cl (**3**).

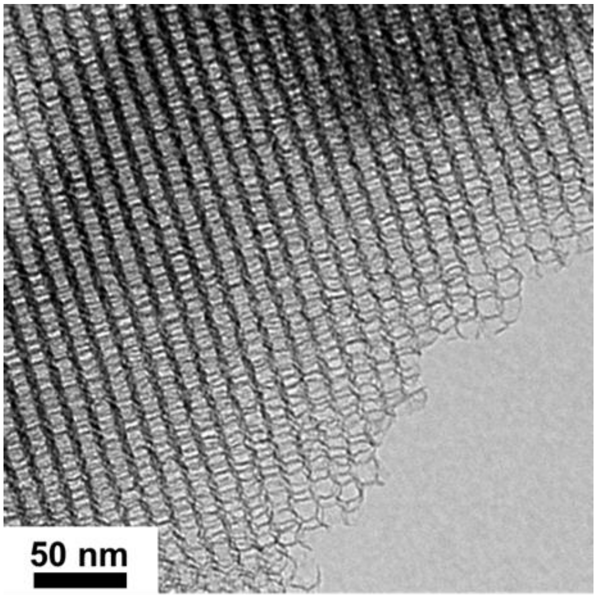

Figure S7. TEM image of OMC after acid etching of Fe_3O_4 NPs**.**

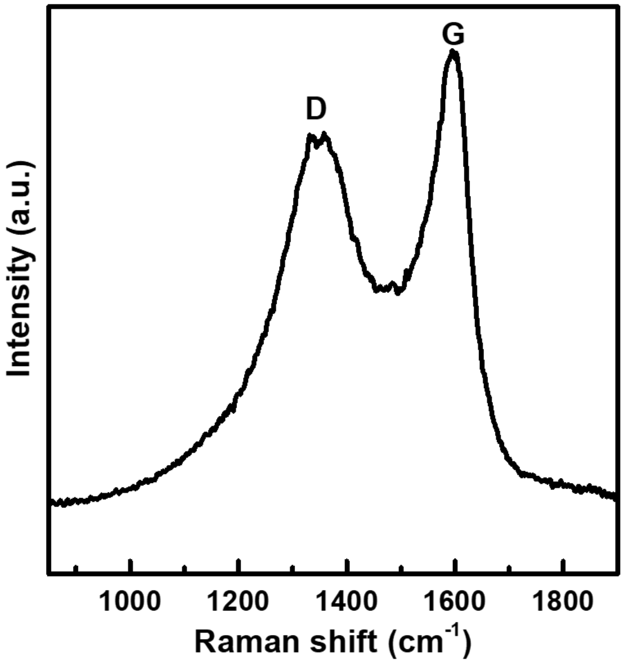

Figure S8. Raman spectrum of OMC after 900 °C treatment.

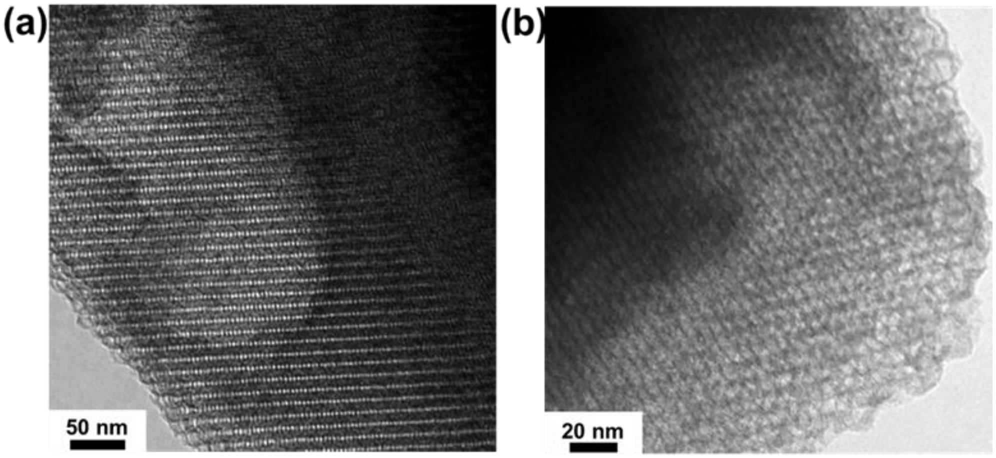

Figure S9. TEM images of Ir complexes loaded on OMC: (a) **Ir-1**; (b) **Ir-3**.

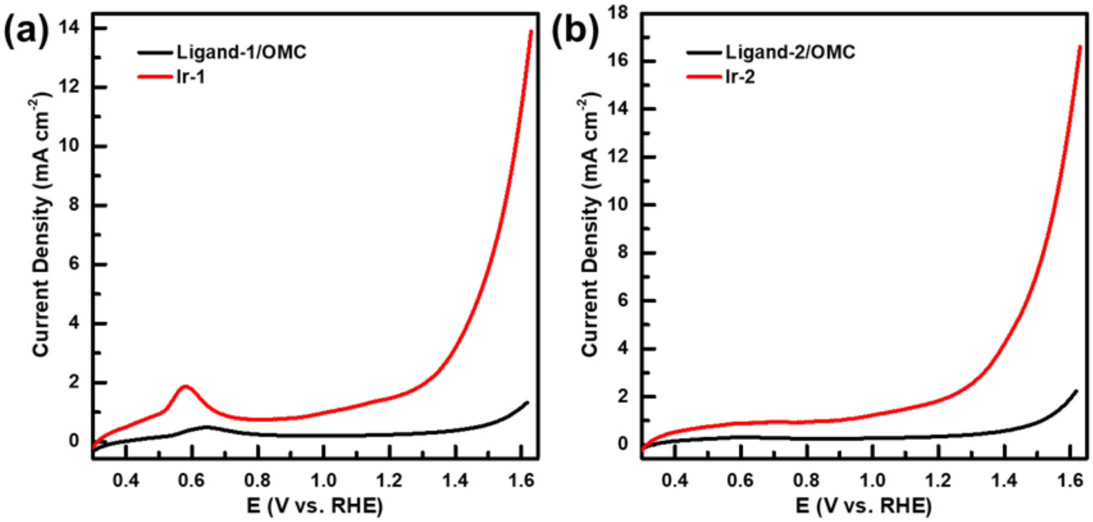

Figure S10. LSV plot of Ir complexes/OMC and L1/OMC: (a) **Ir-1** and L1/OMC; (b) **Ir-2** and L1/OMC.

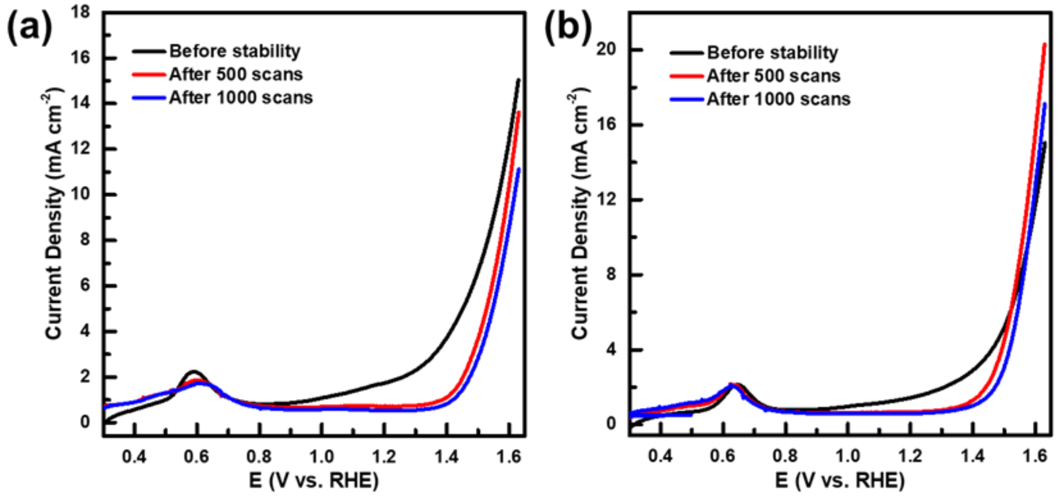

Figure S11. Stability test of Ir complexes/OMC catalysts with continuous LSV test: (a) **Ir-1**; (b) **Ir-3**.

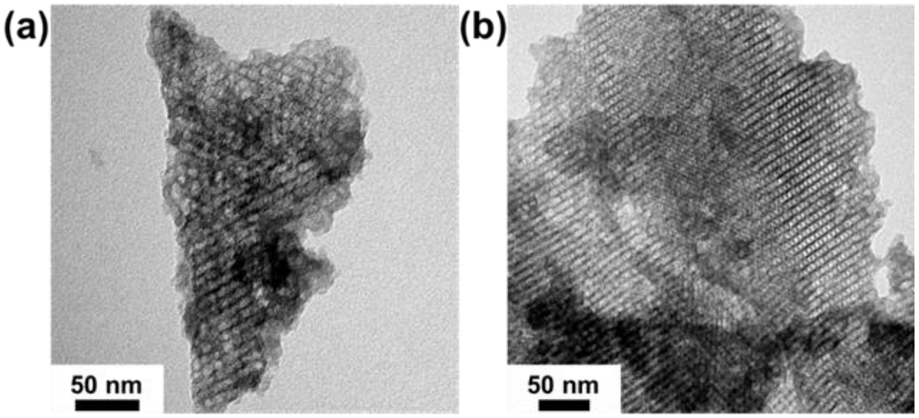

Figure S12. TEM images of Ir complexes/OMC after stability tests: (a) **Ir-1**; (b) **Ir-3.**

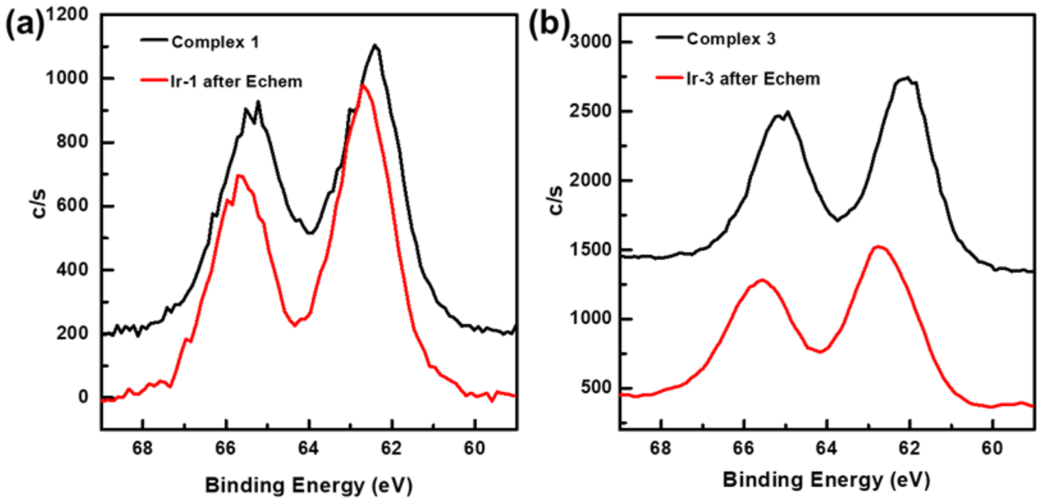

Figure S13. (a) XPS spectra of complex **1** and **Ir-1** after electrochemical test; (b) XPS spectra of complex **3** and **Ir-3** after electrochemical tests.

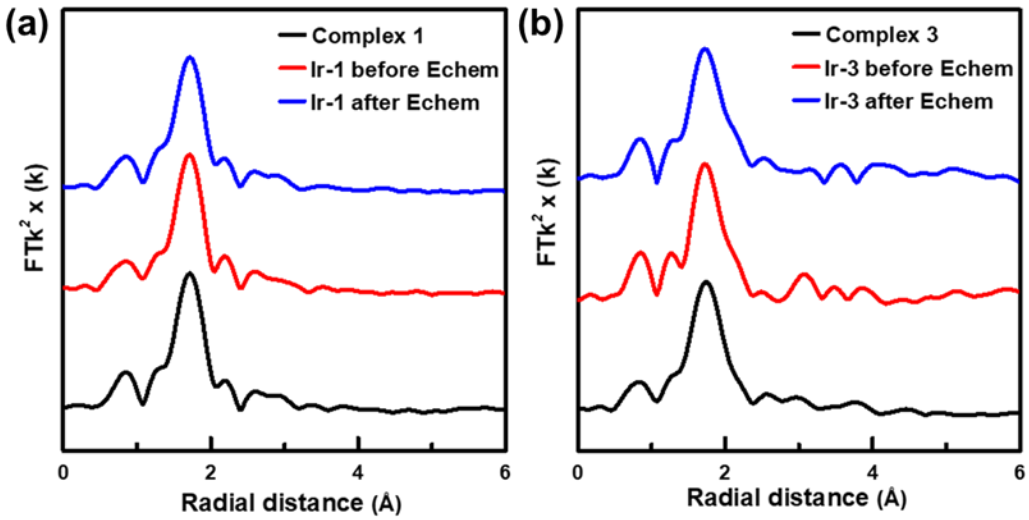

Figure S14. (a) Ex-situ EXAFS analysis of complex **1** and **Ir-1** for before and after electrochemical tests; (b) ex-situ EXAFS analysis of complex **3** and **Ir-3** for before and after electrochemical tests.

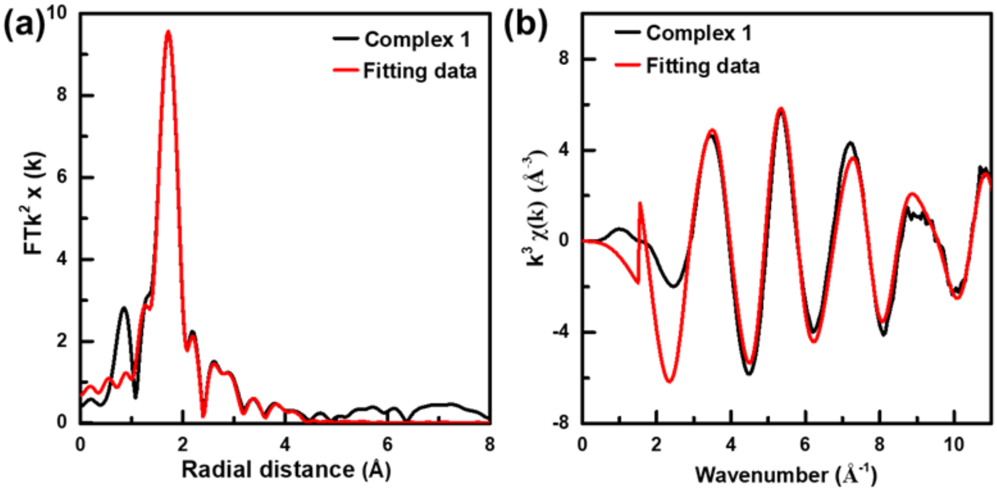

Figure S15. Ir L-edge EXAFS spectra fittings for as-synthesized complex **1**. (a) Fitting in R-space; (b) Fitting in k-space.

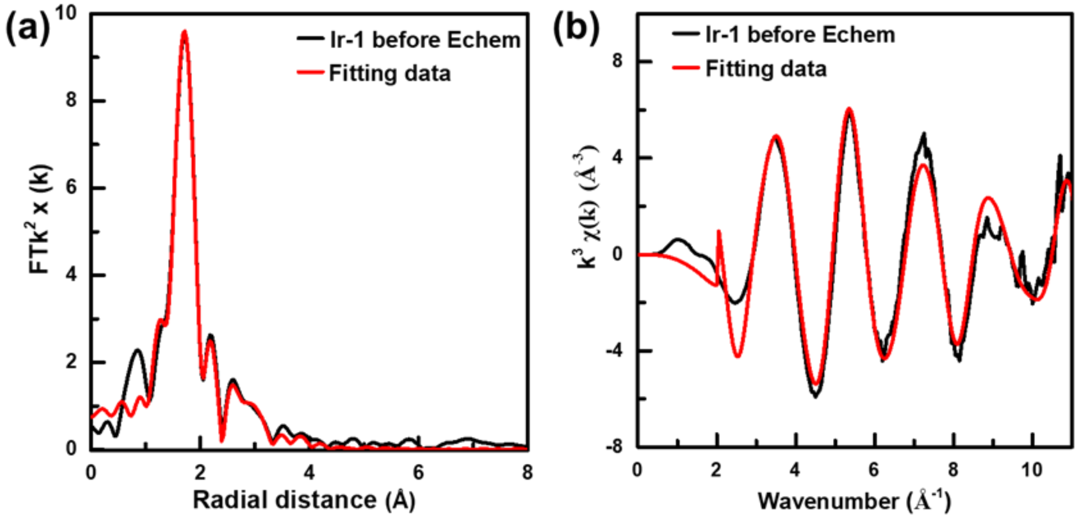

Figure S16. Ir L-edge EXAFS spectra fittings for **Ir-1** before electrochemical tests. (a) Fitting in R-space; (b) Fitting in k-space.

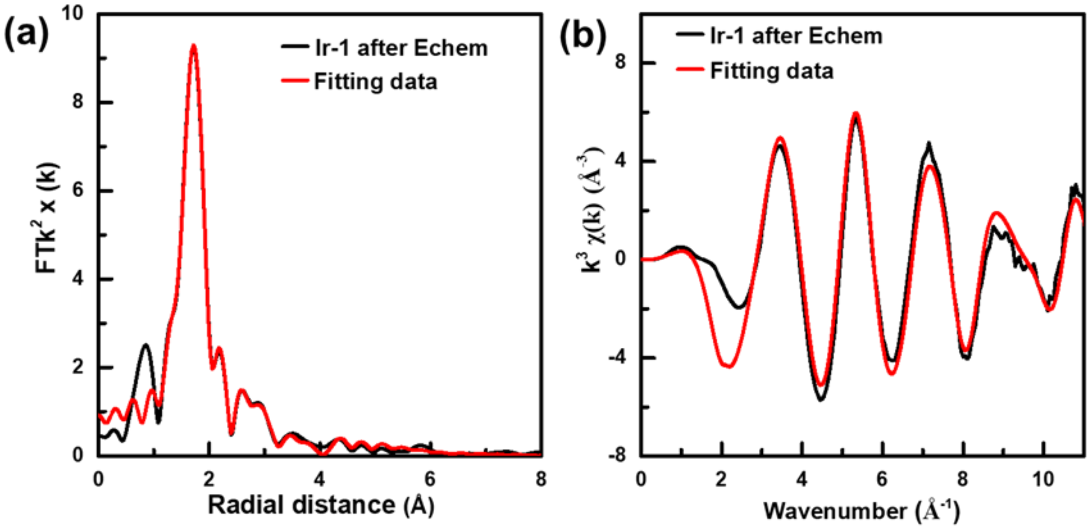

Figure S17. Ir L-edge EXAFS spectra fittings for **Ir-1** after electrochemical tests. (a)Fitting in R-space; (b) Fitting in k-space.

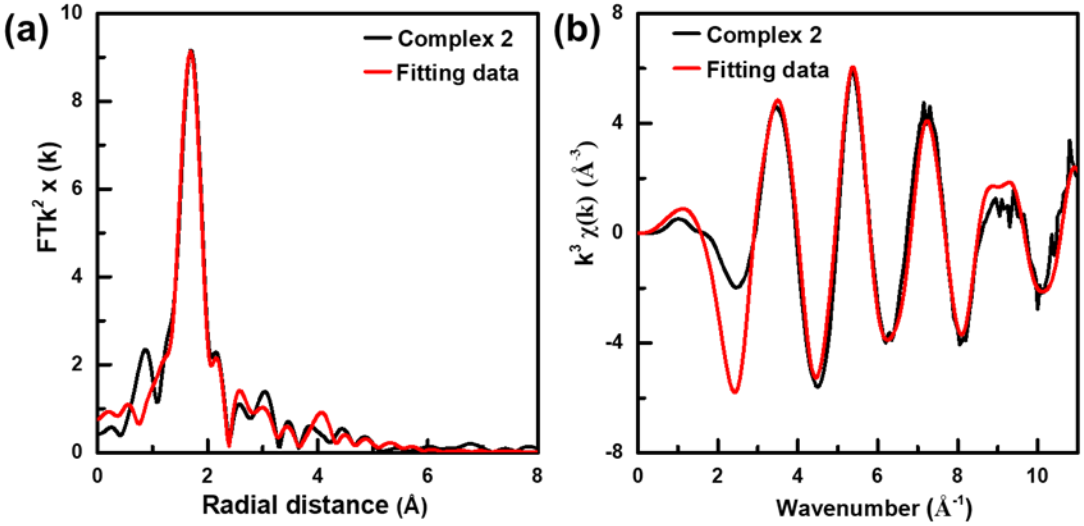

Figure S18. Ir L-edge EXAFS spectra fittings for complex **2**. (a) Fitting in R-space; (b) Fitting in k-space.

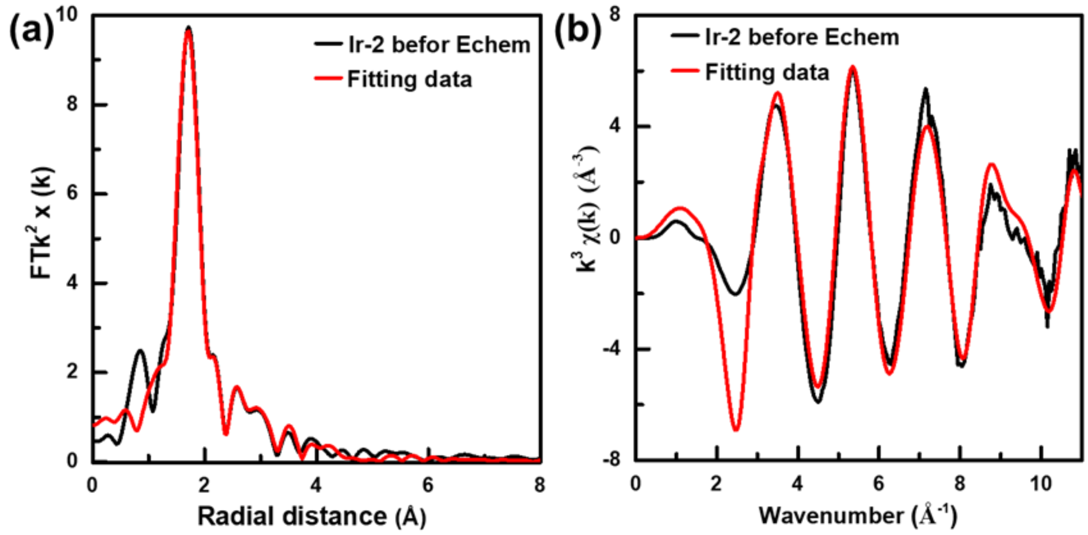

Figure S19. Ir L-edge EXAFS spectra fittings for **Ir-2** before electrochemical tests. (a) Fitting in R-space; (b) Fitting in k-space.

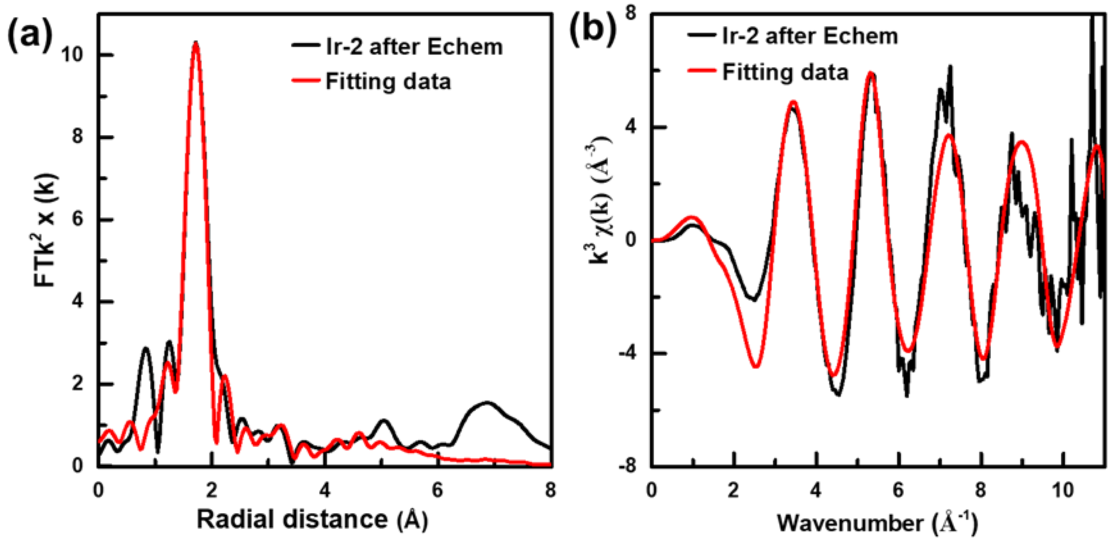

Figure S20. Ir L-edge EXAFS spectra fittings for **Ir-2** after electrochemical tests. (a) Fitting in R-space; (b) Fitting in k-space.

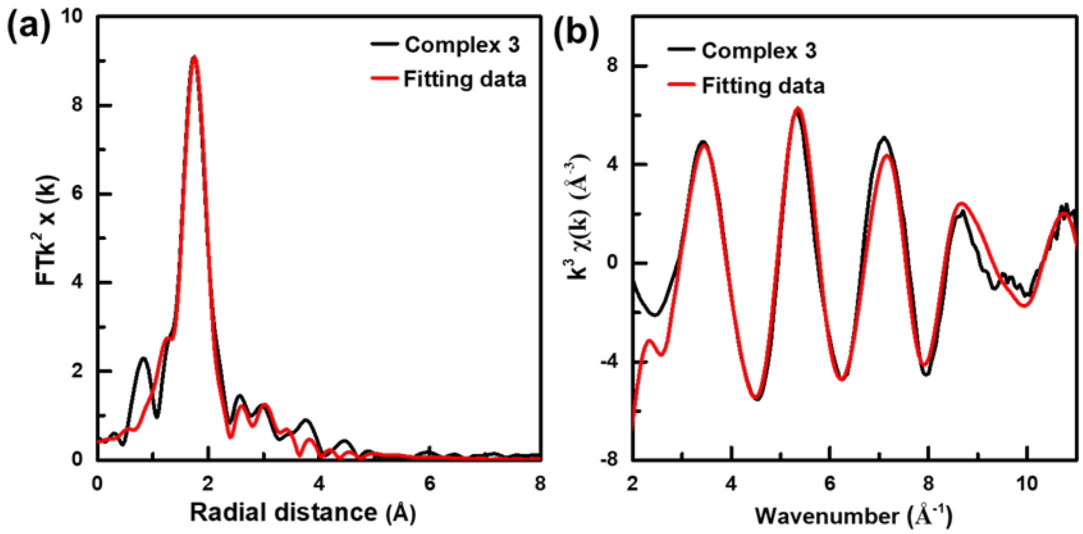

Figure S21. Ir L-edge EXAFS spectra fittings for complex **3**. (a) Fitting in R-space; (b) Fitting in k-space.

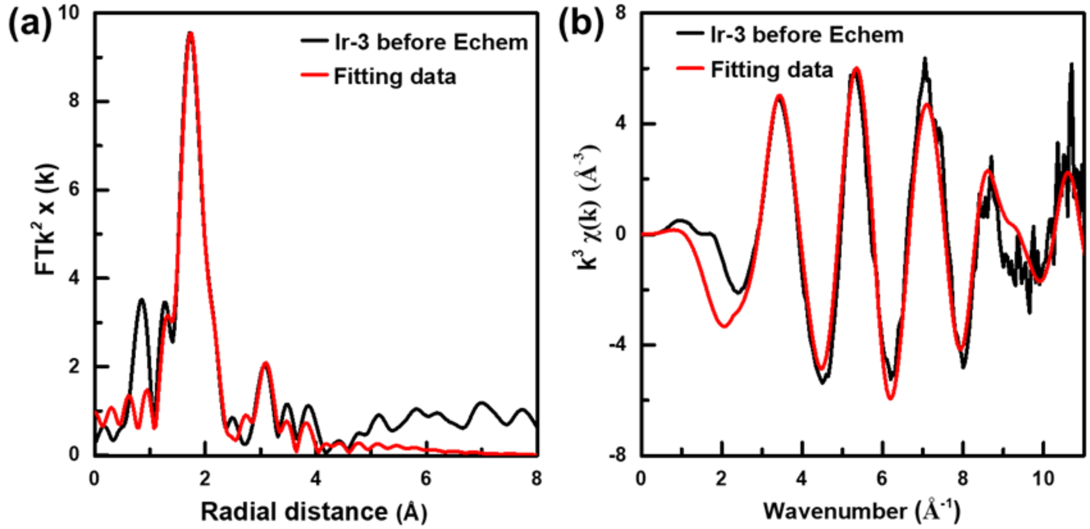

Figure S22. Ir L-edge EXAFS spectra fittings for **Ir-3** before electrochemical tests. (a)Fitting in R-space; (b) Fitting in k-space.

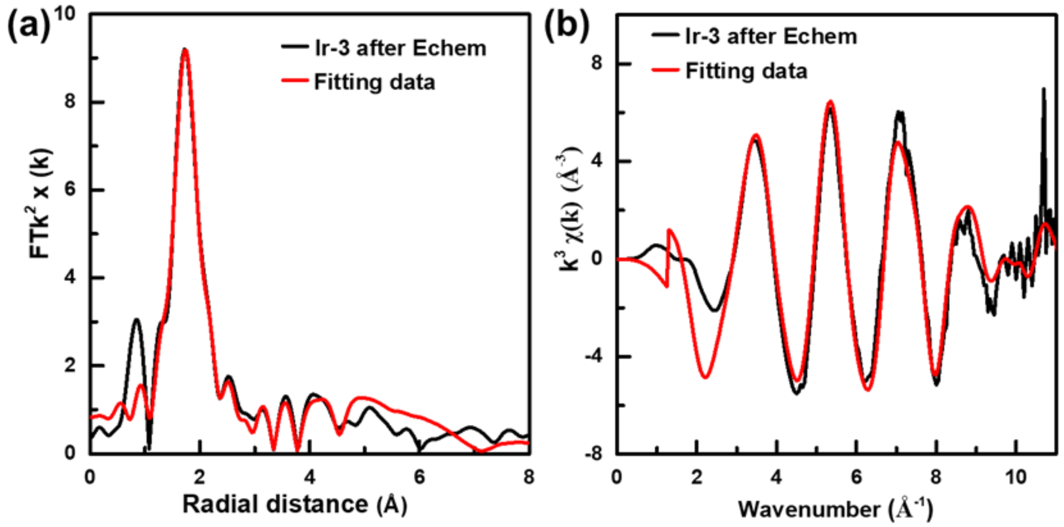

Figure S23. Ir L-edge EXAFS spectra fittings for **Ir-3** after electrochemical tests. (a) Fitting in R-space; (b) Fitting in k-space.

Calculation Details

We examined the binding of the Ir complexes to a periodic sheet of graphene using PBE-D3 DFT. The graphene sheet was a 6x6 supercell containing 72 carbon atoms in a 14.8 x 14.8 x 20.7 Å triclinic cell. We optimized the 3 Ir structures near and far from the graphene surface. The binding energy was calculated as the difference in energy between the near and far-optimized structures. After geometry optimizations of the Ir structures on the graphene sheet, the largest cross-sections of the Ir structures were superimposed with the graphene sheet. We then counted the number of graphene atoms engulfed by the Ir structures' cross-sections (while also accounting for van-der-Waals' radii). The footprint was defined as the total number of graphene atoms required to bind each of the Ir complexes.

We also probed the binding of the Ir structures to a low-density (2.46 gcc) amorphous carbon (LDAC) surface generated using ReaxFF reactive dynamics.[1] This carbon surface was formed via heating and quenching of carbon, such that the final density is ~246 gcc. The carbon surface consists of 754 atoms in a 33.5 x 33.5 x 30.7 Å periodic box. The LDAC has >790 atoms and therefore required a multiscale reactive force field simulation since the number of atoms is not practical with DFT. Universal Force Field molecular dynamics was established by us with an accuracy close to QM DFT.[2] Since the surface area is large and the geometry is irregular, we calculated the binding of 4 Ir molecules onto surface and estimated the average per-molecule binding energy as the total binding energy divided by 4. Similar to graphene, we optimized the Ir complexes near and far from the surface, and calculated the binding energy as the energy difference of these two optimizations.

Reference:

[1] M. D. Hossain, Q. Zhang, T. Cheng, W. A. Goddard III, Z. Luo, *Carbon*, submitted for publication.

[2] A. K. Rappe, C. J. Casewit, K. S. Colwell, W. A. Goddard, W. M. Skiff, *J. Am. Chem. Soc.* **1992**, *114*, 10024-10035.
